# *All-in-one* exome sequencing approach for genetic testing of unexplained premature ovarian insufficiency

**DOI:** 10.1093/hropen/hoag058

**Published:** 2026-06-17

**Authors:** Anu Valkna, Triin Kikas, Ülle Jakovlev, Oliver Mõttus, Avirup Dutta, Külli Erlang, Margus Punab, Kristiina Rull, Maris Laan

**Affiliations:** Chair of Human Genetics, Institute of Biomedicine and Translational Medicine, University of Tartu, Tartu, Estonia; Chair of Human Genetics, Institute of Biomedicine and Translational Medicine, University of Tartu, Tartu, Estonia; Centre of Endocrinology, Clinic of Internal Medicine, East-Tallinn Central Hospital, Tallinn, Estonia; Chair of Human Genetics, Institute of Biomedicine and Translational Medicine, University of Tartu, Tartu, Estonia; Chair of Human Genetics, Institute of Biomedicine and Translational Medicine, University of Tartu, Tartu, Estonia; Women’s Clinic, East-Tallinn Central Hospital, Tallinn, Estonia; Chair of Human Genetics, Institute of Biomedicine and Translational Medicine, University of Tartu, Tartu, Estonia; Andrology Clinic, Tartu University Hospital, Tartu, Estonia; Department of Surgery, Institute of Clinical Medicine, University of Tartu, Tartu, Estonia; Women’s Clinic, Tartu University Hospital, Tartu, Estonia; Women’s Clinic, Institute of Clinical Medicine, University of Tartu, Tartu, Estonia; Chair of Human Genetics, Institute of Biomedicine and Translational Medicine, University of Tartu, Tartu, Estonia

**Keywords:** premature ovarian insufficiency, genetic etiology, exome sequencing, monogenic infertility, copy number variant, microdeletion, multidisciplinary management

## Abstract

**STUDY QUESTION:**

Is exome sequencing (ES) an efficient approach for simultaneous analysis of causative single gene defects and copy number variants (CNV) in unexplained premature ovarian insufficiency (POI)?

**SUMMARY ANSWER:**

Among 51 idiopathic POI cases, a conservative molecular diagnostic yield of 12% was achieved with equal contribution of pathogenic or likely pathogenic (P/LP) monogenic causes and pathogenic microdeletions.

**WHAT IS KNOWN ALREADY:**

POI is a clinically and genetically heterogeneous condition, yet most cases remain idiopathic. Although numerous monogenic causes and CNVs have been implicated in POI, standardized clinical guidelines for comprehensive genetic analysis are still lacking.

**STUDY DESIGN, SIZE, DURATION:**

This observational study of 51 unexplained POI cases implemented an *all-in-one* ES-based approach to analyze P and LP variants in 288 POI candidate genes and large (>0.5 Mb) pathogenic CNVs leading to POI.

**PARTICIPANTS/MATERIALS, SETTING, METHODS:**

Patients were recruited and phenotyped at two tertiary care women’s health and endocrinology centers in Estonia. Bioinformatic processing of ES data and assessment of rare causal variants in POI were performed using an in-house analysis pipeline including automated filtering and manual assessment for pathogenicity, followed by experimental validation via Sanger sequencing (monogenic findings) and chromosomal microarray analysis (CNVs).

**MAIN RESULTS AND THE ROLE OF CHANCE:**

Six of 51 unexplained POI cases had confident molecular findings, including three cases with P/LP monogenic variants and three carrying large pathogenic CNVs. Overall molecular diagnostic yield was estimated to be 12% (6 of 51). There was a statistically significant overrepresentation of likely causal genetic findings in primary amenorrhea (5 of 9 cases, 56%) compared to secondary amenorrhea/oligomenorrhea (1 of 42 cases, 2%) (*P *= 3.0 × 10^−4^). Primary amenorrhea cases presented diverse findings—TP63 p.(Arg97Gly) in three sisters (including one identified by cascade screening), 15q25.2 microdeletion in two unrelated subjects (POI relevant haploinsufficient genes: *CPEB1, BNC1*), and NR5A1 p.(Gly328Arg) in one case. A secondary amenorrhea case with ∼8.5 Mb microdeletion at Xq27.3–Xq28 enabled us to narrow down a critical region for non-recurrent Xq terminal losses causing POI (relevant haploinsufficient genes *FMR1*, *AFF2*, *CETN2*, *HAUS7*, and *EMD*). Additionally, heterozygous variants *TBX6* c.622-2A>T, *EXO1* c.136del, and NR2F2 p.(Val307Ala), as well as 1q21.1 microdeletion (two carriers), 1p36.22, and 12q21.1 microduplications were classified as potentially interesting Variants of Uncertain Significance (VUS) that require validation before their causal link to isolated POI can be assigned. *TBX6* c.622-2A>T and 1q21.1 del have been implicated in other primary phenotypes, Mayer–Rokitansky–Küster–Hauser syndrome (MRKH, Müllerian anomalies), and 1q21.1 microdeletion syndrome (MIM #612474), respectively. A novel variant NR2F2 p.(Val307Ala) was identified in an isolated oligomenorrhea patient; the same substitution was independently detected in an unrelated male subject presenting oligozoospermia and unilateral cryptorchidism. No autosomal recessive POI cases were identified in our cohort, supporting a high level of heterogeneity and population-specificity in the genetic etiologies of POI, likely shaped by diverse demographic histories.

**LARGE SCALE DATA:**

All variants linked identified in this study have been submitted to the NCBI ClinVar database (https://www.ncbi.nlm.nih.gov/clinvar/) and FerGI (https://www.eshre.eu/Specialty-groups/Special-Interest-Groups/Reproductive-Genetics/FeRGI) databases.

**LIMITATIONS, REASONS FOR CAUTION:**

All recruited participants were of white European ancestry and living in Estonia. Thus, the results might not apply to other ethnic groups. This study was conducted in a relatively small and well-selected cohort. Validation in larger and more diverse cohorts is needed to further assess the utility of ES in solving idiopathic POI cases.

**WIDER IMPLICATIONS OF THE FINDINGS:**

The study’s findings support the efficient use of ES as a comprehensive, *all-in-one* genetic test to achieve molecular diagnosis in unexplained POI, capturing both monogenic variants and pathogenic CNVs. Expanded genetic testing in POI is conceptually and clinically justified to reduce idiopathic cases and enable timely personalized management of reproductive and general health, including cascade testing of at-risk relatives. Given that ES enables the detection of both monogenic variants and CNVs, stepwise strategies may no longer be the most optimal, underscoring an urgent need to develop a standardized pipeline and guidelines for the generation, analysis, and interpretation of NGS data as well as for the counseling and management of patients based on their molecular findings.

**FUNDING:**

This study was funded by the Estonian Research Council grants PRG1021 and PRG3041 (to M.L.).

**DISCLOSURES:**

The authors declare no conflicts of interest.

WHAT DOES THIS MEAN FOR PATIENTS? Primary ovarian insufficiency (POI) is a condition in which the ovaries lose their normal function before 40 years of age, leading to irregular or absent menstrual periods and difficulties achieving pregnancy. Currently, two in three POI cases remain unexplained. There is an emerging knowledge that different genetic factors can contribute to POI. In this study, we developed an improved genetic testing approach for POI that combines the analysis of both small-scale (single-gene) and large-scale (parts of chromosomes) abnormalities. In piloting this approach on 51 unexplained POI cases, causative genetic findings were identified in 12% of cases, with both single-gene changes and chromosomal microdeletions contributing similarly to the condition. Women who had never started menstruation were more likely to receive a genetic diagnosis than women whose menstruation stopped later in life. The study highlights the value of comprehensive genetic testing in women with POI, helping to improve diagnosis, guide patient care, and identify at-risk family members who may benefit from genetic counseling and testing.

## Introduction

The average age of natural menopause is approximately 50 years in the general population (InterLACE Study Team, 2019). However, up to 3.5% women experience premature menopause (premature ovarian insufficiency, POI) before the age of 40 (Li *et al.*, 2023). POI is a clinically heterogeneous disease that can present as primary amenorrhea, characterized by the absence of menarche or oligomenorrhea/secondary amenorrhea, referring to abnormal menstrual patterns in individuals with prior menses. Causes of POI are heterogeneous, including iatrogenic (6–47% of cases), autoimmune (5–17%), genetic (10–30%), and environmental (1%) factors; however, the etiology in many cases remains poorly understood, as 36–67% of cases remain idiopathic (Stuenkel and Gompel, 2023).

High heritability and familial clustering of POI indicate a substantial genetic contribution, with marked etiological heterogeneity (Silvén *et al.*, 2022; Verrilli *et al.*, 2023). X-linked chromosomal abnormalities are the most common genetic cause identified in both primary and secondary POI cases, including Turner syndrome (45, X) with its mosaic forms, and structural variants (e.g. Xq microdeletions and Xp22.12 microduplication) (Panay *et al.*, 2024). Also, some recurrent pathogenic autosomal microdeletions (e.g. 15q25.2 and 10q26.3) and microduplications (e.g. 6q21) have been found as high-impact risk factors to POI (McGuire *et al.*, 2011; Tšuiko *et al.*, 2016). However, only a few systematic genome-wide screens aimed at discovering and characterizing copy number variants (CNVs) contributing to POI have been performed, with variable classification of CNV pathogenicity (McGuire *et al.*, 2011; Tšuiko *et al.*, 2016; Jolly *et al.*, 2019; Heddar *et al.*, 2022).

Among monogenic causes of POI, the *FMR1* gene premutation (55–200 CGG repeats in the 5′ regulatory region) is the most widely recognized single-gene contributor. It is associated with RNA-mediated toxicity, repeat-associated translation of potentially toxic proteins, mitochondrial dysfunction, and impaired mitosis, which collectively contribute to disease pathogenesis (Rose and Brown, 2020). The past decade has brought along an expansion in the understanding of monogenic forms of POI, driven by the widely available exome-sequencing (ES) approach, enabling analysis of all coding genes simultaneously. Hundreds of candidate genes for monogenic forms of POI have been proposed, implicated in various biological pathways such as gonadal development, DNA repair and genome integrity, folliculogenesis, and mitochondrial metabolism. The molecular diagnostic yield of ES in POI has been reported to range from 16% to 60% (Heddar *et al.*, 2022; Ke *et al.*, 2023; Vogt *et al.*, 2024; McGlacken-Byrne *et al.*, 2025). However, methodological limitations in research have challenged the utility of this approach in routine clinical settings e.g. sampling bias due to consanguinity or enrichment of familial cases, possible publication bias in preferentially reporting cases with findings, variable classification of variant pathogenicity, lack of validated gene–disease relationships (GDR), and improper consideration of inheritance modes (Tucker *et al.*, 2022b; Riera-Escamilla *et al.*, 2023). To address inconsistencies in interpreting phenotype–genotype links, the Female Reproductive Genetics Initiative (FeRGI) consortium has initiated a systematic assessment of GDR in POI, incorporating standardized variant classification and expert reviews (Female Reproductive Genetics Initiative (FeRGI), 2024). Currently, 223 GDR have been evaluated in POI.

Present international guidelines include chromosomal analysis (karyotyping, chromosomal microarray (CMA), fluorescence in situ hybridization (FISH), or PCR-based methods), and *FMR1* premutation testing as part of the standard diagnostic evaluation for POI (Panay *et al.*, 2024). While these approaches can successfully identify established genetic causes of POI (including large CNVs when using CMA or targeted FISH), they may not capture the full spectrum of causal genetic variants. The recent guideline also recommends next-generation sequencing (NGS) as an optional tool for extended genetic testing in POI (Panay *et al.*, 2024). However, standardized workflows and criteria for its implementation are lacking beyond adherence to both ESHRE (Matthijs *et al.*, 2016) and the American College of Medical Genetics and Genomics (ACMG) guidelines for the classification of monogenic variant pathogenicity (Richards *et al.*, 2015). Using NGS data for CNV analysis is not routinely recommended.

This study aimed to develop and test an *all-in-one* ES-based approach in analyzing the genetic causes of POI. As a significant innovation, the developed ES data analysis pipeline included parallel assessment of pathogenic (P) or likely pathogenic (LP) variants in 288 POI candidate genes and a genome-wide screen for large (>0.5 Mb) CNVs, potentially affecting the dosage of critical genes and/or chromosomal stability. This comprehensive approach resolved 6/51 cases of idiopathic POI, underscoring ES as an underutilized resource for efficient and cost-effective detection of diverse monogenic and structural variants leading to POI.

## Materials and methods

### Ethics statement

The study was approved by the Ethics Review Committee of Human Research of the University of Tartu, Estonia (permission no. 361T-25 with last amendment 401/M-5). Written informed consent was obtained from each patient before recruitment to evaluate and use their clinical data for scientific purposes. The study was conducted in accordance with the Helsinki Declaration.

### Formation, phenotyping, and clinical characteristics of the study group

The FEMale Reproductive cohort of Estonia (FEMARE) has been recruited at the two largest hospitals in the country, specializing in women’s healthcare—The Women’s Clinic of Tartu University Hospital (TUH), Tartu, and the Women’s Clinic and Centre of Endocrinology of East-Tallinn Central Hospital (ETCH), Tallinn (Supplementary Materials and methods). POI was diagnosed based on international guidelines—a loss of ovarian activity before 40 years of age, with disordered menstrual cycles (spontaneous amenorrhea or oligomenorrhea) for at least 4 months, and an elevated FSH concentration (>25 IU/l, reference values are available in Supplementary Table S1) (Panay *et al.*, 2024). Cases diagnosed during routine clinical workup with known non-genetic (e.g. severe gynecological trauma or operation, chemo- or radiotherapy, autoimmune disorder) or genetic causes (chromosomal aberration, *FMR1* premutation) of POI were excluded from the current study. Patients who met the inclusion criteria were invited to participate in the study by their managing clinician. All participants underwent comprehensive interviews at the recruitment, during which detailed clinical histories and family medical background were collected. All participants were of European origin and living in Estonia.

The study group comprised 51 cases with unexplained, non-syndromic POI (median age at recruitment: 37 years; range 18–55 years), including one pair of sisters (Table 1). Seventeen patients (33%) reported comorbidities considered unrelated to POI manifestation, including hypothyroidism in five cases. Patients had observed POI-related symptoms at a median age of 25 (range 13–38) years. Nine women (18%) had been diagnosed with primary amenorrhea; most cases, 42 of 51 (82%), presented secondary amenorrhea/oligomenorrhea. Half of the patients (n = 25) were nulligravida, and the other half (n = 26) had experienced at least one pregnancy. Successful delivery was achieved in 20 cases, with a median age of 25.5 years (range 18–34). There were no statistically significant differences in live births between pregnancies achieved by using ART (5/14; 36%) compared to non-ART pregnancies (15/37; 41%).

**Table 1.** Clinical characteristics of the FEMARE cohort.

Parameters	Full cohort	PA	SA
	n = 51	n = 9	n = 42
**General parameters [median (5th–95th percentile)]^#^**			
Age at recruitment (years)	37 (19–52)	34 (18–43)	37.5 (24–51)
Self-reported age of first symptoms (years)	25 (14–36)	16 (15–17)	29 (14–36)*
Height (cm)	168 (159–178)	171 (163–176)	167 (159–177)
Weight (kg)	67 (48–100)	70 (56–75)	66.5 (50–97)
FSH (IU/l)	65.8 (27–139.7)	71.5 (39.4–130.9)	65.2 (29–138)
**Pregnancy outcomes [number of patients (%)]**			
Nulligravida	25 (50%)	5 (56%)	20 (48%)
Achieved pregnancy	26 (51%)	4 (44%)	22 (52%)
Live birth	20 (39%)	3 (33%)	17 (40%)
Spontaneous	15 (75%)	1 (33%)	14 (82%)
Autologous ART	2 (10%)	0	2 (12%)
Donor-oocyte ART	3 (15%)	2 (67%)	1 (6%)
Age at first live birth (years)	25 (18–34)	31 (27–34)	24 (20–32)

# Medians with 5th–95th percentile ranges were calculated for the full cohort and for the subgroups.

All clinical parameters between primary (PA) and secondary amenorrhea/oligomenorrhea (SA) cases were compared using either the Mann–Whitney *U* test (continuous variables) or Fisher’s exact test (categorical variables).

* *P *= 0.003; statistically significant difference after Bonferroni correction for multiple testing (α = 0.05/12 = 0.0042).

### ES data generation and bioinformatic processing

Blood samples were collected in a clinical setting, and genomic DNA was extracted in the research laboratory using the QIAamp DNA Blood Maxi Kit (QIAGEN GmbH, Hilden, Germany) according to the manufacturer’s instructions. ES library preparation, data generation by NGS service laboratories, and an in-house developed pipeline for bioinformatic processing of the sequencing data have been previously described in detail (Juchnewitsch *et al.*, 2024; Lillepea *et al.*, 2024; Valkna *et al.*, 2025). ES and subsequent bioinformatic processing of reads were performed either at the Genomics unit of the Institute for Molecular Medicine Finland (FIMM), Helsinki, Finland (40 samples) or the accredited laboratory of the Department of Laboratory Genetics, Genetics and Personalized Medicine Clinic, TUH (11 samples) (Supplementary Materials and methods). Variant Call Format (VCF) files of each sample were generated using sequencing reads aligned to the human genome reference (GRCh38). VCF files were filtered for variant quality using identical parameters (exclusion DP < 10 and GQ < 20) and merged into a single VCF file using bcftools (v1.14) (Danecek *et al.*, 2021). Variant annotation was carried out by the Ensembl Variant Effect Predictor (VEP; v111; McLaren *et al.*, 2016) in the offline mode using a predetermined set of flags and plugins (Supplementary Table S2). The distribution of ES reads across chromosomes was assessed to confirm that common aneuploidies had not been missed during routine diagnostic workup (Supplementary Materials and methods). The workflow of the ES data processing and analysis is presented in Supplementary Fig. S1.

### The formation of the POI candidate genetic panel

The list of candidate genes was compiled from several databases and literature resources. Firstly, we used expert-reviewed gene panels from the Genomics England PanelApp (ENG; https://panelapp.genomicsengland.co.uk/) and PanelApp Australia (AUS; https://panelapp-aus.org/). The POI gene panel included 67 (ENG, ver. 1.71) and 162 genes (AUS, ver. 0.393), respectively (date accessed 10.01.2026). We also assessed gene panels for differences in sex development (DSD), including 67 (ENG, ver 4.12) and 139 genes (AUS, ver 1.33), respectively (date accessed 10.01.2026) (Supplementary Fig. S2, Supplementary Table S3). Secondly, the OMIM database (https://www.omim.org/; date accessed 10.01.2026) was screened with the term “premature ovarian failure”. Further female infertility candidate genes were retrieved from a recent systematic review by Van Der Kelen *et al.* (2023). Inheritance modes linked to each gene were derived from the source data.

The total number of POI candidate genes screened for P/LP variants was 288. One hundred and forty genes in the panel were uniquely linked to POI, 123 to DSD, and 25 to both phenotypes. Half of the genes (145 of 288) were implicated in autosomal recessive (AR) conditions, followed by autosomal dominant (AD) conditions (87 of 288). Thirty-three genes were reported to have either monoallelic or biallelic inheritance modes (AD/AR), and 16 had X-linked inheritance. For seven genes, the inheritance mode was not provided by the source, and they were analyzed as AD/AR. The analyzed gene panel of 288 genes included 197 genes (68% of the panel) listed in the FeRGI female infertility candidate gene list, with GDR classified as definitive (20 genes), strong (22 genes), moderate (37 genes), limited (99 genes), and no evidence/not evaluated (19 genes) (Supplementary Table S3; https://www.eshre.eu/Specialty-groups/Special-Interest-Groups/Reproductive-Genetics/FeRGI; 16 February 2026, date accessed). All FeRGI genes for POI with moderate, strong, and definitive evidence were included in the gene list analyzed in this study (Supplementary Table S4).

### Filtering and pathogenicity assessment of rare monogenic variants in the analyzed genes

Custom-designed, predetermined filters were applied to VEP output files to automatically exclude variants with an unlikely causal effect (common, synonymous, or deep intronic) (Supplementary Table S5). Variants with overall minor allele frequency (MAF) >1% in the gnomAD database (v4.1.0; https://gnomad.broadinstitute.org/) and Combined Annotation Dependent Depletion (CADD) score (https://cadd.gs.washington.edu/) lower than 10 were excluded from further assessment.

Online AI-based platforms Franklin by Qiagen (https://franklin.genoox.com/clinical-db/home) and Varsome (https://varsome.com/) were used for the initial evaluation of variant pathogenicity. Variants predicted to be likely benign (LB) or benign (B) were excluded from further steps. For cases with heterozygous P/LP variants in genes linked to AR forms of POI, unfiltered ES data were further examined to potentially identify a second P/LP variant that may have been excluded by the stringent variant filtering pipeline.

All variants predicted as P/LP, or VUS by both AI platforms, were manually analyzed following the recommendations of the American College of Medical Genetics and Genomics (ACMG) (Richards *et al.*, 2015) and the updated recommendations by the ClinGen Sequence Variant Interpretation (SVI) Working Group (https://clinicalgenome.org/working-groups/sequence-variant-interpretation/). The final pathogenicity assessment considered *in silico* predictions, literature, and database records, and collected clinical data.

**Figure 1.**
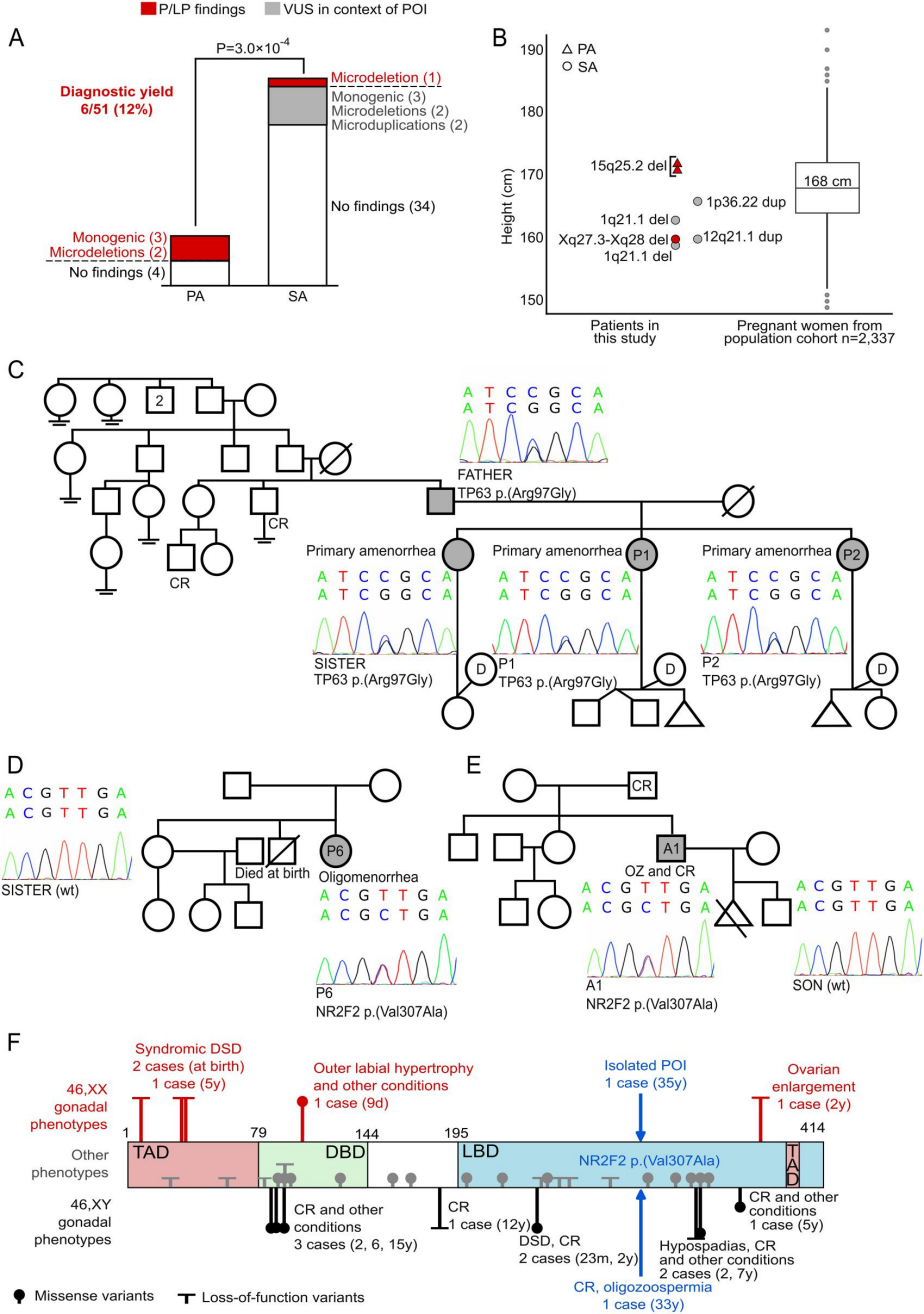
**Overview of all genetic findings and the context of TP63 p.(Arg97Gly) and NR2F2 p.(Val307Ala) variants**. (**A**) Comparative findings in primary (PA) and secondary amenorrhea/oligomenorrhea (SA) patients. (**B**) Comparative height of patients with genetic findings in this study and an Estonian population-based cohort of pregnant women (n = 2,337; Supplementary Materials and methods). (**C**) Pedigree of sisters with primary amenorrhea and carrying TP63 p.(Arg97Gly) variant. P1 and P2 were analyzed using ES data, whereas their sister and father were identified as carriers in cascade screening. (**D**) Pedigree of oligomenorrhea case P6 with carrying NR2F2 p.(Val307Ala) variant. (**E**) Pedigree of an Estonian infertile man A1 presenting oligozoospermia (OZ) and cryptorchidism (CR), identified as a carrier of NR2F2 p.(Val307Ala) in an independent study aiming to screen monogenic causes of male infertility (O. Mõttus, M. Laan, unpublished data; Supplementary File S1). (**F**) Overview of literature cases identified with *NR2F2* missense or loss-of-function (nonsense, frameshift, and canonical splice site alterations) variants classified as P/LP. Variants identified in the current study are highlighted in blue. References to 36 variants described in clinical cases and additional data about their phenotype are available in Supplementary Table S12. d, days; DBD, DNA-binding domain; del, deletion; dup, duplication; DSD, differences in sex development; ES, exome-sequencing; LBD, ligand-binding domain; m, months; P/LP, pathogenic/likely pathogenic; POI, premature ovarian insufficiency; TAD, transactivation domain; VUS, variant of unknown significance; y, years; wt, wild-type.

All retained variants passed a visual inspection for the quality of sequencing reads using the Integrative Genomics Viewer (IGV) software (Robinson *et al.*, 2023), and low-confidence variant calls were discarded. All predicted P/LP variants were confirmed by Sanger sequencing, whereas heterozygous AR variants were visually inspected using IGV only. PCR primers are listed in Supplementary Table S6, chromatograms of validated variants are shown in Fig. 1 and Supplementary Fig. S3, and IGV plots for heterozygous AR variants are shown in Supplementary Figs S4, S5, and S6.

### Genome-wide screen and targeted validation of large microdeletions and microduplications

The primary sequencing analysis and CNV calling using ES data were performed by the service provider using the established pipeline based on the Illumina DRAGEN Bio-IT Platform (v3.9 and v3.10, GRCh38 assembly for FIMM ES data and v4.1.7 or v4.3.13 for TUH ES data). The analysis considered high-quality deletions and duplication calls (>30 quality score) from the VCF file, excluding common (>3 carriers) and <0.5 Mb CNVs. The IGV software was used to visualize read quality, and low-confidence calls were omitted (Robinson *et al.*, 2023). The remaining CNVs were annotated using AnnotSV (Geoffroy *et al.*, 2023) and StrVCTVRE (Sharo *et al.*, 2022). Variant pathogenicity was estimated using the StrVCTVRE algorithm (cut-off score >0.37) and the ACMG class provided by AnnotSV (≥3). Recurrent pathogenic microdeletions and microduplications were defined based on the ClinGen database (https://clinicalgenome.org). Prioritized recurrent (n = 4) and non-recurrent (n = 3) CNVs were validated by chromosomal microarray analysis (CMA) at the accredited service laboratory of the Genetics and Personalized Medicine Clinic, TUH. CMA was implemented using SNP genotyping data from Infinium Global Diversity Array-8 v1.0 BeadChip, containing ∼1.8 million markers and the Illumina iScan system. CNVs were called using the Genome Studio v2011.1. CMA results are available in Supplementary Figs S7, S8, S9, S10, S11, S12, and S13.

### Additional investigations based on genetic analysis results

Exome-wide rare variant profiles of patients identified as carriers of the same P/LP variant were compared pairwise to assess the presence (kinship coefficient > 0.088) or absence (close to 0) of relatedness (Supplementary Table S7).

For a subset of patients with P/LP variants, follow-up assessments were available and conducted upon patient consent to gather additional clinical and family health data. When possible, cascade screening was offered to family members, who would benefit from immediate genetic counselling and testing (P1, P2, P6). DNA from family members was collected using a buccal swab from the Swab Collection and DNA Preservation System (Norgen Biotek Corp., Thorold, ON, Canada) and extracted using the Norgen Biotek Microbiome DNA Isolation Kit (Norgen Biotek Corp.) following the manufacturer’s instructions. Sanger sequencing chromatograms are shown in Fig. 1.

## Results

### Overview of the findings in FEMARE idiopathic POI cases

Likely causal findings were identified in 6 of 51 study subjects, including heterozygous variants TP63 p.(Arg97Gly) (two cases) and NR5A1 p.(Gly328Arg), as well as high-confidence pathogenic microdeletions 15q25.2 del (1.61 Mb, two cases) and Xq27.3–Xq28 del (8.51 Mb) (Fig. 1A, 1B, Tables 2 and 3, Supplementary Tables S8, S9, and S10). The molecular diagnostic yield was estimated 12%. There was a significant overrepresentation of findings in primary (5 of 9) vs. secondary amenorrhea/oligomenorrhea (one of 42) cases (55.6% vs. 2.4%; Fisher’s exact test; OR = 51.3, 95% CI 4.7–553.7; *P *= 3.0 × 10^−4^). Patients with NR5A1 p.(Gly328Arg) and 15q25.2 del remained nulligravida; the other two microdeletion carriers had experienced natural conceptions and deliveries (at 26–32 years) after hormone replacement therapy (HRT), whereas both TP63 p.(Arg97Gly) carriers achieved motherhood using donor oocytes and ART.

**Table 2.** Heterozygous variants in autosomal dominant genes in unexplained POI cases.

	Variant data, evidence, and interpretation				Patients’ clinical data		
Patient code(age at onset^a^/recruitment)	Gene; cDNA(protein change)rs-no Transcript	MAF CADD REVEL^b^	Previous genotype–phenotype data of the variant	ACMG (2015) ClinGen (2025) NCBI ClinVar **Final** ^c^	H (cm) W (kg) BMI FSH (IU/l)^d^	Reproductive phenotype Other reported conditions	Other health conditions
P1 (15/39y)	*TP63*: c.289C>G p.(Arg97Gly) n/a NM_003722.5	n/a 25.0 0.63	Novel variant; alternative missense substitutions have been reported in isolated POI p.(Arg97Pro) (Tucker *et al.*, 2022a) and ectrodactyly p.(Arg97Cys) (Zenteno *et al.*, 2005).	LP VUS not reported **LP**	175 72 23.5 76.9	Primary amenorrhea; received HRT; ART pregnancies with donor oocytes, resulting in one delivery at 34 y and one miscarriage at 38 y.	Hypoplastic skin, hypopigmentation on the arms, thin and brittle hair, nail dysplasia, agenesis of three wisdom teeth (Fig. 1C).
P2 (17/35y)	Sister of P1, see above				168 79 24.8 38.1	Primary amenorrhea; received HRT; ART pregnancies with donor oocytes, resulting in miscarriage at 30 y and delivery of twins at 31 y.	Hypoplastic skin, hypopigmentation on the arms, thin and brittle hair, nail dysplasia, dacryostenosis (Fig. 1C).
P3 (17/18y)	*NR5A1*: c.982G>A p.(Gly328Arg) rs1832340912 NM_004959.5	1.7 × 10^−6^ 29.8 0.96	46, XY case with partial virilization of external genitalia (Song *et al.*, 2018).	LP LP P (46, XY DSD) **P**	171 72 24.6 114.1	Primary amenorrhea; received HRT; **nulligravida**; no ART procedures.	Isolated POI, no other major health problems.
P4 (16/33y)	*TBX6:* c.622-2A>T rs780739101 NM_004608.4	8.5 × 10^−7^ Splice AI score 1^e^	MRKH, total Müllerian aplasia, ovarian aplasia (Sandbacka *et al.*, 2013).	P P not reported **VUS** (isolated POI)	166 60 21.7 n.a	Secondary amenorrhea; received HRT; **nulligravida; ** no ART procedures.	Not available for the follow-up assessment.
P5 (31/32)	*EXO1*: c.136del p.(Leu46*) rs750915959 NM_130398.4	5.9 × 10^−6^ 33 not applicable	Novel variant; heterozygous variant in *EXO1* previously reported in isolated POI (Luo *et al.*, 2020).	P LP LP (disease n.a.) **VUS** (isolated POI)	177 73.9 23.5 42.2	Oligomenorrhea; received HRT; **nulligravida**; no ART procedures.	Isolated POI, no other major health problems.
P6 (35/37y)	*NR2F2:* c.920T>C p.(Val307Ala) n/a NM_021005.4	8.5 × 10^−7^ 32 0.97	Novel variant; *NR2F2* is confidently linked with 46, XX and 46, XY DSD and gonadal dysgenesis phenotypes (Fig. 1F).	VUS VUS not reported **VUS** (isolated POI)	167 67 24.0 65.5	Oligomenorrhea; received HRT; **nulligravida**; no ART procedures.	Isolated POI, no other major health problems. (Fig. 1D).

a Age of onset reported by the patient.

b MAF (minor allele frequency) of non-Finnish Europeans (NFE) is based on the gnomAD database v4.1.0 (https://gnomad.broadinstitute.org/); Combined Annotation dependent depletion (CADD) score based on GRCh38-v1.6 (https://cadd.gs.washington.edu/); REVEL scores were sourced from Franklin by Qiagen (https://franklin.genoox.com/clinical-db/home).

c The final classification of variants was based on the ACMG guidelines (Richards *et al.*, 2015) and the ClinGen SVI group recommendations (https://clinicalgenome.org/working-groups/sequence-variant-interpretation/), considering also the collected clinical data and entries by others in the NCBI Clinvar database, (https://www.ncbi.nlm.nih.gov/clinvar/). Detailed variant pathogenicity assessment is in Supplementary Tables S8 and S9.

d Retrospective data for serum FSH measurements at variable timepoints during clinical diagnosis or management; age at FSH measurement unavailable.

e For splice variants, SpiceAI Delta score (range 0–1) was assessed using an AI-based tool (https://spliceailookup.broadinstitute.org/); pathogenicity cut-offs are the following: 0.2 (high recall), 0.5 (recommended), and 0.8 (high precision).

DSD, differences in sex development; H, height; HRT, hormone replacement therapy; LP, likely pathogenic; MRKH, Mayer–Rokitansky–Kuster–Hauser syndrome; n/a, not available; P, pathogenic; POI, premature ovarian insufficiency; VUS, variant of uncertain significance; W, weight; y, years.

**Table 3.** Identified recurrent disease-causing microdeletions and novel large (>500 kb) deletions and duplications.

	Variant data, evidence, and interpretation		Patients’ clinical data		
Patient code(age at onset^a^/recruitment)	CNV ID^b^Coordinates and size (hg38)^c^OMIM ID/ClinGen ID Population prevalence	Variant pathogenicity classification and data in the literature	H (cm) W (kg) BMI FSH (IU/l)^d^	Reproductive phenotype	Family health history
P7 (16/46y)	15q25.2 del (recurrent) Chr15:82552258–84403399 1.85 Mb (P7) Chr15:82550590–84163390 1.61 Mb (P8) MIM #614294/ISCA-37500 (HI score 3^e^) 1:29,085 (Coe *et al.*, 2014)	15q25.2 microdeletion syndrome (**P**). Mild to moderate DD/ID, neuropsychiatric conditions, seizures; short stature, hypotonia, and craniofacial abnormalities; malformations of the upper limb, chest, abdomen, heart, and urinary system; anemia; POI in women, cryptorchidism in men (McGuire *et al.*, 2011; Tšuiko *et al.*, 2016).	171 68 23.2 n.a.	Primary amenorrhea; induced menarche at 16 y; received HRT; ectopic pregnancy at 23 y; natural conception and deliveries at 26 and 28 y; no ART procedures.	One child with epilepsy; no other suspected POI cases in the family.
P8 (16/25y)			172 74 25 139.9	Primary amenorrhea; induced menarche at 16 y; received HRT; **nulligravida**; no ART procedures. Other^f^—surgically corrected malocclusion of teeth; psoriasis.	Grandmother’s sister—late menarche; grandmothers’s half-sister—oligomenorrhea; father—leukemia.
P9 (30/45y)	Xq27.3–Xq28 del (non-recurrent) X: 147912179–156010409 8.51 Mb	Microdeletion encompassing *FMR1* (**P**). Large deletions at Xq27.3–Xq28 are mostly in females with ID/DD, mild dysmorphic features, amenorrhea, POI (Fig. 2C; Supplementary Table S13).	160 71 27.7 67.8	Secondary amenorrhea; received HRT; spontaneous conception and delivery with placental abruption at 32 y; two elective pregnancy terminations (age n/a); no ART procedures.	Sister—late menarche (16 y), ectopic pregnancy, and medical termination of gestation with FGR fetus; maternal family—female infertility and pregnancy complications (Fig. 2A).
P10 (31/33y)	1q21.1 del (recurrent) Chr1:146872717–148353326 1.48 Mb (P10) Chr1:146872717–148353641 1.48 Mb (P11) MIM #612474/ISCA-37421 (HI score 3^e^) 1:2,626	1q21.1 microdeletion syndrome (**P**). GDR of isolated POI classified as **VUS**. Variable penetrance and expressivity, including asymptomatic cases (Bourgois *et al.*, 2024). Cognitive, neurological, and psychiatric conditions, DD/ID; microcephaly, dysmorphism, and short stature; heart defects, cataract, hearing loss; abnormalities of the genitalia and/or urinary system (Edwards *et al.*, 2021)^.^; infertility (Bernier *et al.*, 2016; Kikas *et al.*, 2023)	163 64 24 n.a.	Secondary amenorrhea; **nulligravida**; no ART procedures.	Sister—oligomenorrhea; maternal uncle—severe psychiatric conditions and heart problems, died at 48 y; maternal cousin—deafness.
P11 (16/30y)			159 50 19.7 139.2	Secondary amenorrhea; received HRT; ART pregnancies with donor oocytes, resulting in spontaneous miscarriages at 25, 27, and 29 y. Other^f^—two hernia operations in childhood.	Sister—suspected oligomenorrhea; sister’s children—mental health problems.
P12 (25/37 y)	1p36.22 dup (recurrent) Chr1:12273027–12776092 0.5 Mb	This microduplication (**VUS**) is located in the proximal end of the critical region for 1p36 microdeletion syndrome, characterized by severe DD/ID (Fig. 2D)	166 60 21.7 55.2	Oligomenorrhea; received HRT; **nulligravida**; no ART procedures.	Maternal family members—ID, DD, and cognitive impairment; half-brother died at birth with an unclear cause; high incidence of cancers in the pedigree (Fig. 2B).
P13 (35/42 y)	12q21.1 dup (non-recurrent) Chr12:76430772–77942978 1.51 Mb	No previous reports on large duplications at 12q21.1 (**VUS**)	160 78 30.4 111.4	Oligomenorrhea; spontaneous conception and delivery at 25 y; no ART procedures.	No family data available.

a Age of onset reported by the patient.

b Recurrent and non-recurrent variants refer to copy number variants (CNV) with repeated or random breakpoints, respectively.

c Genomic coordinates of microdeletions and microduplications based on chromosomal microarray analysis (CMA) data; experimental validation of all CNVs using CMA is presented in Supplementary Figs S7, S8, S9, S10, S11, S12, and S13.

d Retrospective data for serum FSH measurements at variable timepoints during clinical diagnosis or management; age at FSH measurement unavailable.

e Haploinsufficiency score from the ClinGen database, whereby an HI score of 3 has a suggested clinical classification of pathogenic.

f Other conditions in the medical history of the patient that are potentially linked to the genetic finding.

DD, developmental delay; FGR, fetal growth restriction; GDR, gene–disease relationship; H, height; HRT, hormone replacement therapy; ID, intellectual disability; P, pathogenic; VUS, variant of uncertain significance; W, weight; y, years.

Heterozygous findings *TBX6* c.622-2A>T, *EXO1* c.136del, NR2F2 p.(Val307Ala), 1q21.1 microdeletion (two cases), 1p36.22 and 12q21.1 microduplications were classified as potentially interesting VUSs that require validation before their causal link to isolated POI can be assigned (Fig. 1A and B, Tables 2 and 3). No biallelic P/LP variants in autosomal or X-linked recessive genes were identified (Supplementary File S1, Supplementary Table S11, Supplementary Figs S4, S5, and S6).

### Findings in the FeRGI gene panel suggest variable expressivity of variants

Four monogenic variants were identified in the FeRGI female infertility gene list with different levels of evidence for GDR: *NR5A1* (definitive), *TBX6* (moderate), *TP63* (limited), and *EXO1* (limited).

The TP63 p.(Arg97Gly), classified as LP, was detected in three sisters with primary amenorrhea. The variant was identified in the discovery ES-based study in patients P1 and P2, whereas the third sister was found to carry the same substitution in cascade screening (Table 2, Fig. 1C). This variant is novel and has not been reported in any human genetic databases or the literature. However, an alternative amino acid substitution at the same position, TP63 p.(Arg97Pro), has been found in two cases with early secondary amenorrhea diagnosed after only one or two recorded menstrual cycles (Tucker *et al.*, 2022a). The three sisters presented with other features typically associated with *TP63* mutations, including dry and hypoplastic skin, freckles, hypopigmentation on the arms, thin and brittle hair, nail dysplasia, tooth agenesis, and dacryostenosis. All sisters were able to achieve pregnancy using donor oocytes and ART (at early 30s), indicative of a normally functioning endometrium. The variant was paternally inherited, and the father’s pedigree suggested several possible cases of female and male infertility across three generations.

A pathogenic variant NR5A1 p.(Gly328Arg) was identified in the case P3 with isolated primary amenorrhea, diagnosed at 17 years (Table 2). The variant was previously reported in a 46, XY DSD case presenting under-virilized genitalia (Song *et al.*, 2018). However, P3 had neither anatomical anomalies (as assessed during routine gynecological examination) nor any general health problems. No other potential infertility cases were identified among her relatives.

A splice variant *TBX6* c.622-2A>T was identified in the case P4 with an early secondary amenorrhea (Table 2). Her menarche occurred at 15 years with initially regular cycles but followed by progressively increasing cycle lengths and amenorrhea at 16 years. Previously, the *TBX6 c.622-2A>T* variant has been reported in two 46, XX individuals with Mayer–Rokitansky–Küster–Hauser syndrome (MRKH) characterized by Müllerian anomalies and ovarian aplasia (Sandbacka *et al.*, 2013). No anatomical anomalies were documented in P4 during routine gynecological examination. Due to a lack of clear GDR, this variant was classified as VUS in the context of POI.

*EXO1* c.136del [p.(Leu46*)], also classified as VUS, was found in case P5 presenting oligomenorrhea at 31 years (Table 2). In the literature, a heterozygous EXO1 p.(Thr52Ser) variant has been reported in POI, with functional studies showing impaired homologous recombination (Luo *et al.*, 2020). In contrast, others have demonstrated that partial loss of *EXO1* function does not necessarily disrupt meiosis (Wang *et al.*, 2022). Thus, the contribution of *EXO1* haploinsufficiency to isolated POI remains uncertain.

### *NR2F2* as a plausible novel candidate gene for female and male infertility

A novel NR2F2 variant, p.(Val307Ala), was identified in P6, who was diagnosed with isolated oligomenorrhea (Fig. 1D, Table 2). Her cycle length at 35 years was documented as around 80 days, and at 37 years, she was still nulligravida. Intriguingly, the same variant was independently identified in an unrelated 33-year-old Estonian oligozoospermic man (total sperm count, 14.7 million/ejaculate) with unilateral cryptorchidism (O. Mõttus and M. Laan, unpublished data; Fig. 1E; Supplementary File S1). *NR2F2* is a pleiotropic gene encoding COUP transcription factor 2, and its alterations have been reported in a broad range of isolated or syndromic developmental phenotypes with variable expressivity and severity, including 46, XX and 46, XY DSD cases, cryptorchidism, and other genital congenital conditions (Fig. 1F, Supplementary Table S12 for 17 references describing 36 variants previously reported in clinical cases). The severity of reproductive phenotypes in 46, XX subjects depends on the location of the involved amino acid (Fig. 1F). P/LP variants affecting the transactivation and DNA-binding domains have been reported in severe phenotypes of genital organs (46, XX DSD, outer labial hypertrophy), whereas variants modulating the ligand-binding domain have been observed only in milder conditions of affected ovarian function (POI, ovarian enlargement). This domain-specific effect is not observed in men (Fig. 1F). Due to a lack of sufficiently clear GDR, NR2F2 p.(Val307Ala) was currently classified as VUS in the context of POI.

### High-confidence prediction of large CNVs from ES data

**Figure 2.**
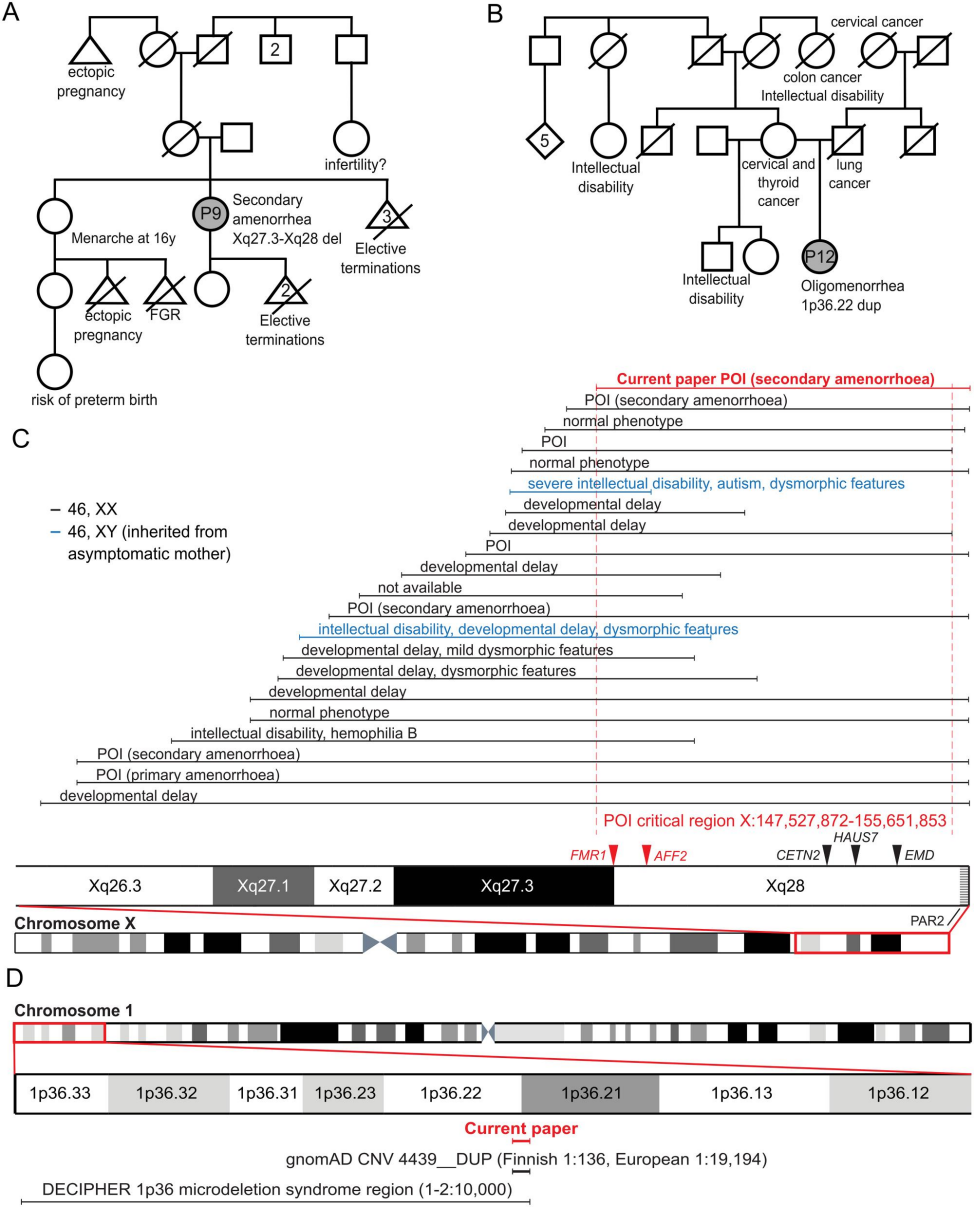
**Context of the Xq27.3–Xq28 microdeletion and 1p36.22 microduplication**. (**A**) Pedigree of a secondary amenorrhea case P9 with a large pathogenic microdeletion at Xq27.3–Xq28 (8.51 Mb). (**B**) Pedigree of an oligomenorrhea case P12 with a recurrent 1p36.22 microduplication (0.5 Mb), classified as Variants of Uncertain Significance (VUS). (**C**) Literature cases with non-recurrent pathogenic microdeletions at Xq27.3–Xq28 and their primary phenotypes (genomic coordinates and references are presented in Supplementary Table S13). Notably, only two male patients, both with intellectual disability and dysmorphic features, have been reported. Chromosomal location of confident and less established premature ovarian insufficiency (POI) genes within the critical microdeletion region are indicated in red and black font, respectively. (**D**) Recurrent 1p36.22 microduplication has an identical proximal breakpoint with the critical region for severe 1p36 microdeletion syndrome that typically occurs *de novo*. According to the gnomAD database (v4.1.0), 1p36.22 microduplication (copy number variant (CNV) 4439_DUP) is enriched in Finland, with a carrier frequency of 1 in 136 subjects, but is rare on a global scale. del, deletion; dup, duplication; FGR, fetal growth restriction; y, years.

Large (≥0.5 Mb) CNVs were identified in seven POI patients (Table 3, Supplementary Table S10). Pathogenic recurrent microdeletions were found at 15q25.2 (P7, P8), and a large non-recurrent POI-linked deletion was detected at Xq27.3–Xq28 (P9, Fig. 2A). Two subjects (P10, P11) carried recurrent pathogenic CNV implicated in 1q21.1 microdeletion syndrome (MIM #612474/ISCA-37421; Edwards *et al.*, 2021); however, so far, the causal link to POI remains unclear. Single cases were identified with microduplications at 1p36.22 (P12, Fig. 2B) and 12q21.1 (P13), classified as VUS.

As an interesting observation, patients with large CNVs presented a trend towards a shorter stature compared to a reference cohort of Estonian pregnant women (median 163 cm vs. 168 cm; permutation test, *P *= 0.1; Fig. 1B). This trend was not observed in POI cases with monogenic findings.

### Microdeletions at 15q25.2 and Xq27.3–Xq28 cause amenorrhea

15q25.2 del was identified in two unrelated patients with POI. Patients with 15q25.2 microdeletions (P7, P8) presented primary amenorrhea. The involvement of 15q25.2 microdeletions to POI has been previously reported in several independent studies (McGuire *et al.*, 2011; Tšuiko *et al.*, 2016; Chen *et al.*, 2020). The deleted region (P9 ∼1.61, P10 ∼1.85 Mb) encompasses 12 protein-coding genes, including *BNC1*, which has been reported to be linked to POI in an autosomal dominant manner (MIM #618723) (Zhang *et al.*, 2018). The critical region also encompasses *CPEB1*, for which haploinsufficiency has been shown to cause POI (Hyon *et al.*, 2016). Recurrent microdeletions at 15q25.2 are rare, with a population prevalence of 1:29 085 (Coe *et al.*, 2014).

We identified a secondary amenorrhea case P9 with ∼8.5 Mb non-recurrent microdeletion at Xq27.3–Xq28 (Fig. 2A, Table 3). A range of large deletions with variable breakpoints at Xq27.3–Xq28 have been reported primarily in females presenting with variable cognitive or reproductive phenotypes, with secondary amenorrhea due to POI being the most common reproductive presentation (Fig. 2C, Supplementary Table S13 for 11 references describing 18 CNVs with variable breakpoints in previously reported clinical cases). P9 sister also had a complicated reproductive history—late menarche (16 years), an ectopic pregnancy, and a terminated gestation due to fetal growth restriction (FGR). Our findings enabled us to narrow down a critical region of Xq terminal deletions responsible for the development of amenorrhea. This region encompasses 114 protein-coding genes, including *FMR1* (FeRGI GDR: definitive) and *AFF2* (limited), as well as the recently proposed candidate genes *CETN2*, *HAUS7*, and *EMD* (Katari *et al.*, 2018; Knight *et al.*, 2024) (Fig. 2C).

### Two secondary amenorrhea cases with recurrent 1q21.1 microdeletions

Recurrent 1q21.1 microdeletions (1.5 Mb) were found in two unrelated secondary amenorrhea cases (P10, P11) with no other major health concerns (Table 3). Most reported patients diagnosed with the 1q21.1 microdeletion syndrome have been children presenting various neurodevelopmental and congenital anomalies; however, incomplete penetrance and variable expressivity of 1q21.1 del have been extensively described (Bernier *et al.*, 2016; Ledig and Wieacker, 2018; Edwards *et al.*, 2021). The deleted region does not include any genes involved in human reproduction, and its exact contribution to POI remains unclear. Still, as the prevalence of 1q21.1 del in the Estonian population is 1:2626 (Männik *et al.*, 2015), the likelihood of observing two cases with this chromosomal alteration among 51 subjects by chance alone is low (exact binomial test; *P *= 1.83 × 10^−4^).

### Oligomenorrhea cases with microduplications at 1p36.22 and 12q21.1 of uncertain significance

P12 with oligomenorrhea diagnosed at 25 years carried a recurrent microduplication (∼0.5 Mb) at 1p36.22 (Fig. 2B, Table 3, Supplementary Table S10), encompassing *VPS13D* and *DHRS3* with no known involvement in reproduction. This variant has a notable prevalence in Finland (1 in 136), a neighboring country to Estonia (Fig. 2D). It was classified as VUS due to insufficient evidence for pathogenicity. Notably, 1p36.22 duplication is located in the proximal end of the critical region for severe 1p36 microdeletion syndrome, characterized by developmental anomalies and intellectual disability (ID) (Jacquin *et al.*, 2023). Several members of the P12 maternal family were reported with ID in three generations (unavailable for cascade screening) (Fig. 2B).

P13 with oligomenorrhea diagnosed at 35 years carried a non-recurrent 1.51-Mb duplication (VUS) at 12q21.1 (Table 3). The only disease-causing gene in this region is *NAV3* (OMIM #621182; pLI = 1; triplosensitivity probability = 0.83), which is partially included in the duplication and is highly expressed in the ovary. No previous links to POI have been reported, and the phenotypic effect of the extra copy remains to be clarified.

## Discussion

This study aimed to evaluate the utility of ES as an *all-in-one* genetic test for molecular diagnostics in POI. To capture the full genetic complexity, we analyzed 288 proposed candidate genes for female infertility in parallel with a genome-wide screen of large CNVs (>0.5 Mb), known to contribute to POI but typically overlooked in ES-based studies. The confident diagnostic yield was 12%; however, gathering further evidence regarding the contribution of identified interesting VUS findings may potentially increase this estimate. Our outcome aligns with prior reports showing that explorative research-based studies, compared to more conservative clinical settings, typically estimate a higher diagnostic yield, which could be achieved when also including genes with limited GDR or variants lacking sufficient clinical validation (Heddar *et al.*, 2022; Ke *et al.*, 2023; Vogt *et al.*, 2024).

Equal representation of monogenic variants and CNVs in our cohort, with three patients in each category, provides support that ES data is suitable for efficient parallel analysis of clinically relevant monogenic and large structural variants. Furthermore, reliable detection of complete or partial deletions of POI-linked genes *STAG3* (3.2 kb), *MND1* (1.4 kb), *FSHR* (105 kb), and *NR5A1* has been reported in cases with early-onset menopause (32 kb) (Jolly *et al.*, 2019; Heddar *et al.*, 2022; Sreenivasan *et al.*, 2022). Restricting ES data utility to monogenic variant detection, as recommended in the current guidelines (Panay *et al.*, 2024), underutilizes its potential added value in infertility workup. As ES can detect both monogenic and certain types of structural variants (large deletions and duplications, single gene losses), integrating CNV analysis into ES-based pipelines should be considered to improve molecular diagnostics of POI. In perspective, given the reduced sensitivity of ES-based CNV calling, a more comprehensive approach using whole-genome sequencing (WGS) could be adopted. However, the current use of WGS in the clinical setting is limited by high cost, data storage requirements, analytical complexity, and challenges in interpreting variants.

Genetic causes were identified in both primary and secondary amenorrhea (Tables 2 and 3). However, in the overall cohort, high-confidence likely causative variants were significantly overrepresented in individuals with primary amenorrhea (Fig. 1A). While genetic factors can underlie both early- and later-onset POI, primary amenorrhea is likely more often associated with a strong genetic contribution, such as single defective genes or highly pathogenic CNVs. Secondary amenorrhea may more frequently reflect complex etiologies, including multigenic and gene–environment interactions, which remain challenging to resolve with current genetic analysis and functional validation frameworks.

A large number of proposed POI candidate genes (Supplementary Fig. S2, Supplementary Table S3) suggests a substantial heterogeneity in the underlying genetic causes. Many POI gene discoveries have arisen from analyzing consanguineous families (França and Mendonca, 2022) or specific populations with enriched variants due to distinct demographic history (Aittomäki *et al.*, 1995). In our cohort, confident P/LP variants in POI were identified in two candidate genes, *NR5A1* and *TP63*, linked to AD forms of infertility. No pathogenic variants were identified in 145 autosomal recessive candidate genes, even under relaxed variant filtering conditions (Supplementary File S1), despite this inheritance form being predominantly reported in the literature. This suggests that the genotype spectrum observed in studies that have been published so far may not apply to all populations, and regional cohorts are expected to have findings only in a fraction of POI genes.

Importantly, variable expressivity and penetrance of P/LP variants in POI-linked genes have been described. For instance, causal molecular findings in *NR5A1* and *TP63* have been reported not only in syndromic developmental conditions but also in isolated POI (Lourenço *et al.*, 2009; Tucker *et al.*, 2019; Jaillard *et al.*, 2020; Mathorne *et al.*, 2020; Huang *et al.*, 2023). In this study, we found three monogenic alterations that have been observed in cases presenting other reproductive phenotypes, *TBX6* c.622-2A>T in 46, XX patients with MRKH syndrome (Sandbacka *et al.*, 2013), NR5A1 p.(Gly328Arg) in a subject with 46, XY DSD (Song *et al.*, 2018) and NR2F2 p.(Val307Ala) in a male case presenting oligozoospermia and cryptorchidism (Fig. 1E, Supplementary File S1). Notably, none of our patients with monogenic findings exhibited severe anatomical abnormalities, supporting incomplete penetrance or variable expressivity of the affected genes and/or specific variants (Table 2). Collectively, these observations indicate that POI could lie along a spectrum of reproductive developmental disorders, extending beyond classic syndromic presentations. Similar observations have been reported for men with low sperm counts (Juchnewitsch *et al.*, 2024; Lillepea *et al.*, 2024).

Two primary amenorrhea cases carried recurrent pathogenic microdeletions at 15q25.2, previously linked to POI (Tšuiko *et al.*, 2016). This region encompasses haploinsufficient genes *BNC1* and *CPEB1*, both implicated as drivers of ovarian dysfunction and POI (Hyon *et al.*, 2016; Zhang *et al.*, 2018). Truncating variants in *BNC1* have also been reported in oligozoospermic men, suggesting an overlapping genetic etiology (Lillepea *et al.*, 2024).

A secondary amenorrhea case was detected with a large loss (8.51 Mb) at Xq27.3–Xq28, a hotspot for non-recurrent microdeletions observed in both isolated and syndromic POI cases (Fig. 2C, Table S13). Notably, the same region is implicated in a rare Xq27.3–Xq28 microduplication syndrome, in which primary testicular failure in males and POI in females, along with growth abnormalities (including FGR), are typical features (Hickey *et al.*, 2013). The exact mechanism by which alterations in this region lead to POI is not fully understood. However, it may include multiple candidate genes linked to POI, such as *FMR1*, *AFF2*, *CETN2*, *HAUS7*, and *EMD* (Fig. 2C). In females, interpretation is further complicated by X-chromosome inactivation, as studies have shown inconsistent and contradictory findings in the context of infertility (Pu *et al.*, 2010; Miranda-Furtado *et al.*, 2018). Additionally, a cluster of genes escaping X-chromosome inactivation is located in Xq28 (Carrel and Willard, 2005), and any structural variant in this region alters the overall genetic dosage, which may also contribute to variable disease phenotype.

In contrast, the large 1q21.1 microdeletion lacks a clearly defined candidate locus implicated in infertility among the hundreds of genes involved. Haploinsufficiency in this region has been reported to cause a low-penetrance syndrome affecting various organs, including the genitourinary tract (Edwards *et al.*, 2021). While the majority of investigated cases have been children, two infertile adult subjects have also been reported, detailing oligozoospermia in one case (Bernier *et al.*, 2016; Kikas *et al.*, 2023). Another possible mechanism is that the large structural change affects the effectiveness of chromosomal recombination in meiosis. This may potentially also apply to rare 1p36.22 and 12q21.1 microduplications identified in this study. Together, these observations underscore the need for research into the mechanisms underlying POI development via structural chromosomal aberrations.

There are several clinical and conceptual justifications for expanded genetic testing in POI. First, the majority of POI cases are secondary and may occur after a period of preserved ovarian function (Panay *et al.*, 2024). This creates a limited window during which women may still conceive or benefit from fertility preservation strategies, underscoring the importance of early risk identification, particularly in the context of increasing maternal age. Genetic testing enables the detection of disease risk before clinical symptoms, thereby facilitating timely reproductive counseling, informed family planning, including early initiation of HRT to optimize reproductive potential, and consideration of options such as oocyte cryopreservation. Second, identification of pathogenic variants in pleiotropic loci is important, as associated non-reproductive phenotypes may be mild yet increase the risk of additional diseases that might otherwise go unrecognized. Also, milder or unnoticed phenotypic manifestations do not exclude complete penetrance in future offspring; for example, broad expressivity has been observed for *NR5A1* mutations and recurrent 1q21.1 microdeletions (Brunetti-Pierri *et al.*, 2008; Eggers *et al.*, 2015; Wang *et al.*, 2017; Kouri *et al.*, 2025). This further highlights the importance of understanding the etiology of POI in each patient. Third, overlapping genetic factors in male and female infertility are commonly acknowledged (Van Der Kelen *et al.*, 2023). Consistently, several variants identified in this study have also been reported in 46, XY subjects with reproductive and genital conditions (Tables 2 and 3). In such contexts, cascade testing may be considered for both female and male family members to assess the risk of genetic infertility. However, interpretation requires careful consideration of variable penetrance and sex-specific expressivity.

In conclusion, our findings support the use of ES as a comprehensive, *all-in-one* genetic test for idiopathic POI, capturing both monogenic variants and large pathogenic CNVs. However, a consensus pipeline and standardized guidelines for interpretation are needed to ensure consistent results. ES also provides opportunities for cascade testing in families with possible genetic infertility, allowing early identification of at-risk relatives. Beyond reproduction, ES may also identify clinically actionable secondary findings, providing additional information relevant to reproductive counseling and individualized assessment of broader health risks (Kasak *et al.*, 2022). Together, these results underscore the value of extended genetic testing in both isolated and syndromic POI.

## Supplementary Material

hoag058_Supplementary_Data

## Data Availability

The data underlying this article are available in the article and in its online supplementary material. All variants identified in this study have been submitted to the NCBI ClinVar database (https://www.ncbi.nlm.nih.gov/clinvar/).
