# Supplementary material for: All-in-one exome sequencing approach for genetic testing of unexplained premature ovarian insufficiency
Source: Hum Reprod Open. 2026 Jun 17;2026(3):hoag058. doi: 10.1093/hropen/hoag058 (PMC13372669; doi:10.1093/hropen/hoag058)
Supplement: hoag058_Supplementary_Data [file hoag058_supplementary_data.zip › HRO-0126.R2_-_Supplementary_information.docx]

Table of Contents

[SUPPLEMENTARY FIGURES 2](#_Toc229816235)

[Supplementary Figure S1. The workflow of the study 3](#_Toc229816236)

[Supplementary Figure S2. Characterization of the gene panel analyzed in this study. 4](#_Toc229816237)

[Supplementary Figure S3. Chromatograms from Sanger sequencing validation of variants in P3, P5, P6. 5](#_Toc229816238)

[Supplementary Figure S4-S6. IGV visualization of heterozygous variants in AR genes 6](#_Toc229816239)

[Supplementary Figure S7-S13. IGV and CMA visualization of CNVs identified in this study 9](#_Toc229816240)

[SUPPLEMENTARY MATERIALS AND METHODS 16](#_Toc229816241)

[Recruitment of participants and retrospective collection of health and family history. 16](#_Toc229816242)

[Biological Sample Collection, DNA Extraction, and Exome Sequencing (ES). 16](#_Toc229816243)

[ES pipeline at the NGS Service Laboratory at FIMM, Helsinki, Finland. 16](#_Toc229816244)

[ES pipeline at the NGS Service Laboratory, TUH, Tartu, Estonia. 17](#_Toc229816245)

[Processing of VCF files and variant annotation using Variant Effect Predictor (VEP). 17](#_Toc229816246)

[Ploidy Estimation. 17](#_Toc229816247)

[Kinship analysis. 18](#_Toc229816248)

[Comparative population based cohort of pregnant women as a reference for population parameters in Figure 1B. 18](#_Toc229816249)

[SUPPLEMENTARY FILE S1 18](#_Toc229816250)

[Assessment of heterozygous P/LP variants in autosomal recessive genes. 18](#_Toc229816251)

[Identification of an independent male patient of NR2F2 p.(Val307Ala) variant (Case A1). 19](#_Toc229816252)

[References 20](#_Toc229816253)

**Supplementary Tables S1-S13 are provided in a separate file (.xlsx).**

# **SUPPLEMENTARY FIGURES**

**
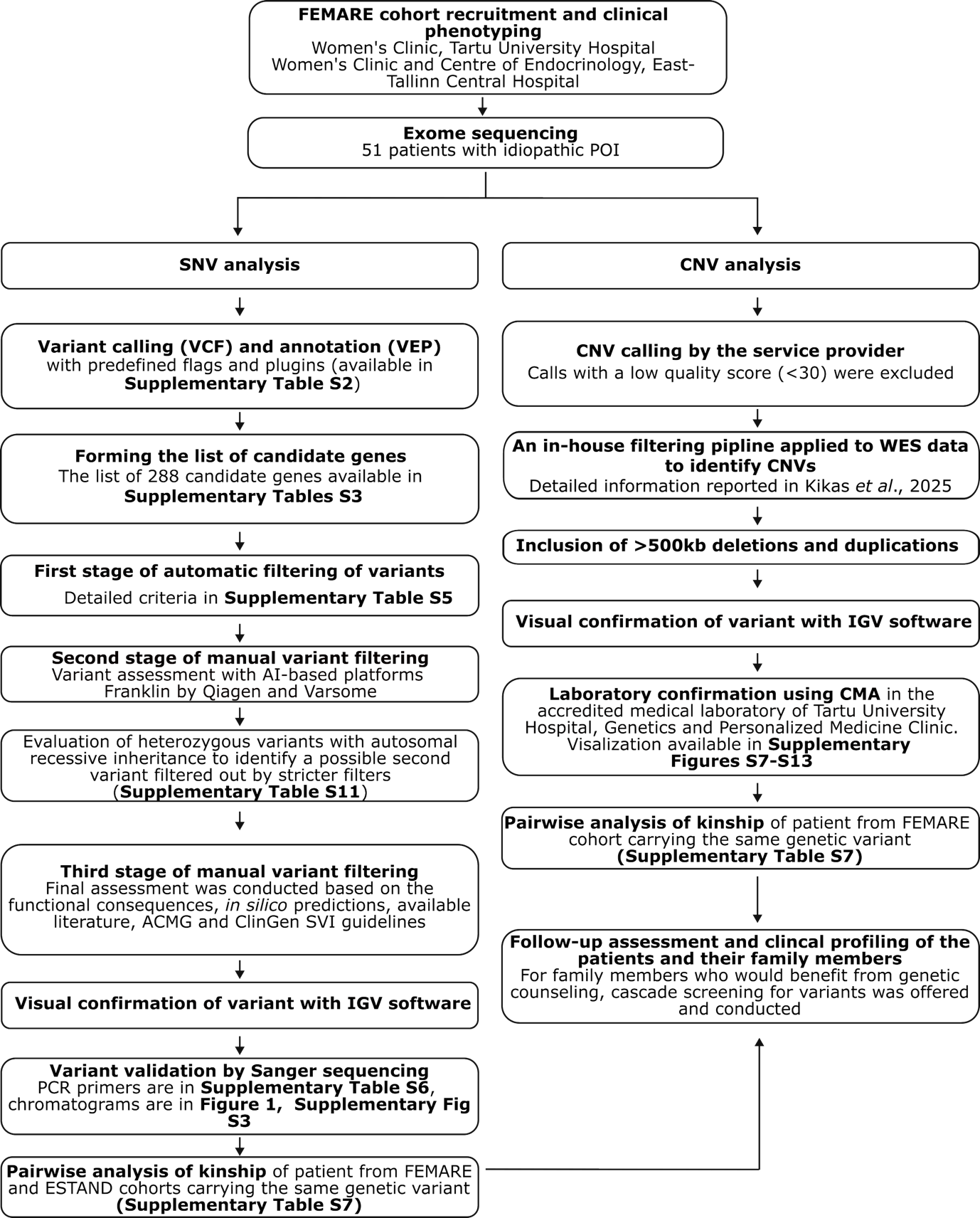
**

**Supplementary Figure S1. *See Figure Legend at the next page.***

## **Supplementary Figure S1. The workflow of the study**

Variant pathogenicity evaluation used ClinVar (https://www.ncbi.nlm.nih.gov/clinvar/) database records and AI-based platform Franklin by Qiagen (https://franklin.genoox.com) and Varsome (https://varsome.com/). The pipeline for CNV analysis has been described previously in detail (Kikas *et al.*, 2025). Both monogenic variants and CNVs were visualized with Integrative Genomics Viewer (IGV) (Robinson *et al.*, 2023). The final manual assessment of variants was based on the American College of Medical Genetics and Genomics (ACMG) guidelines (Richards *et al.*, 2015) and ClinGen Sequence Variant Interpretation (SVI) Working Group (<https://clinicalgenome.org/working-groups/sequence-variant-interpretation/>; date accessed 10.01.2026) (**Supplementary Table S9).** All reported monogenic variants were experimentally validated by Sanger sequencing (**Figure 1, Supplementary Figure S3**) and CNVs by chromosomal microarray analysis (**Supplementary Figures S7-S13).** Supplementary Tables S1-S13 are provided in a separate file (.xlsx).

CMA, chromosomal microarray analysis; CNV, copy number variant; ESTAND, The ESTonian ANDrology cohort; FEMARE, The FEMale Reproductive cohort of Estonia cohort; POI, premature ovarian insufficiency; SNV, single nucleotide variant; SVI, Sequence Variant Interpretation; VCF, variant call format; VEP, Variant Effect Predictor.

**
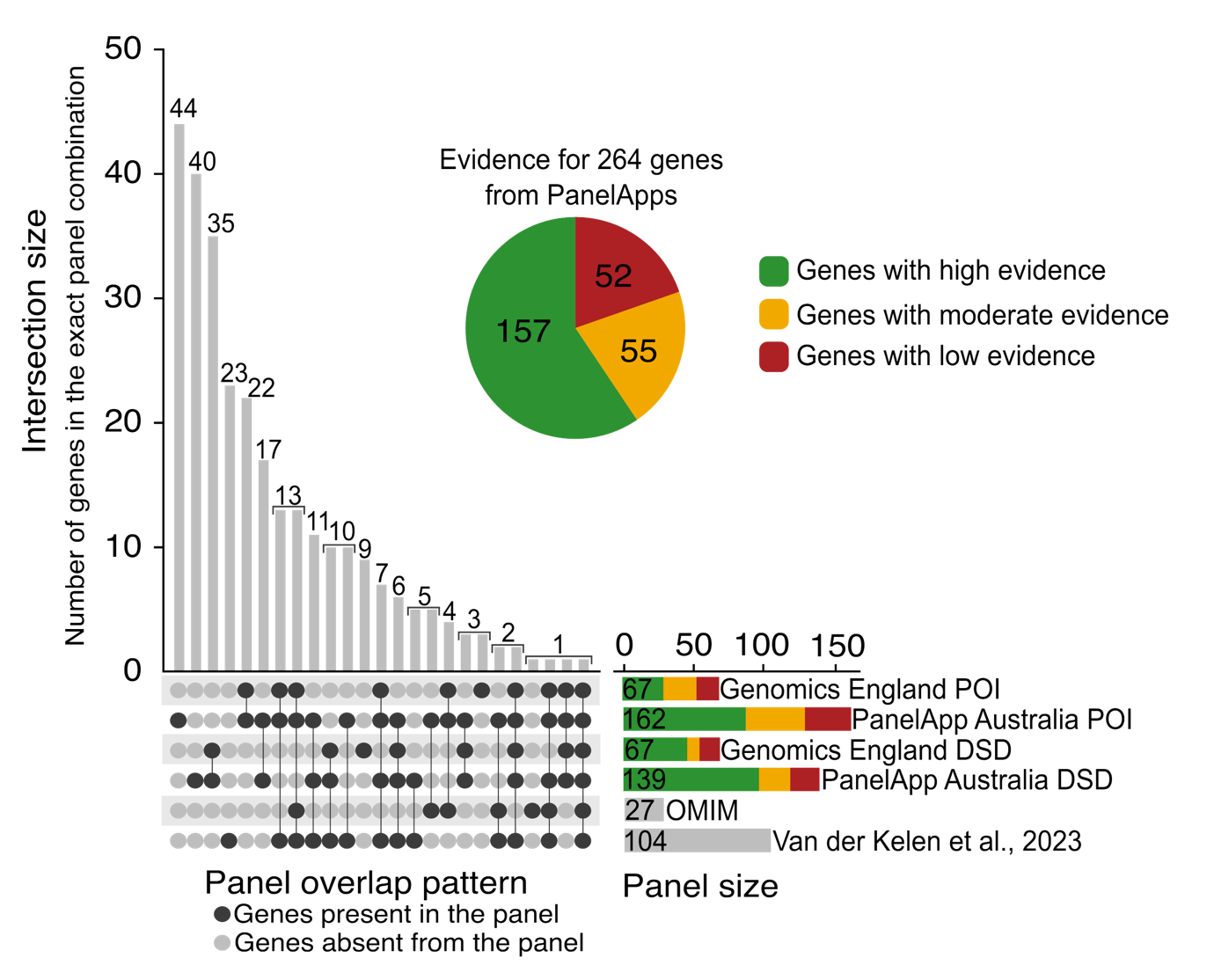
**

## **Supplementary Figure S2. Characterization of the gene panel analyzed in this study.**

The gene panel analysed in this study, comprising 288 candidate genes, was compiled from five independent sources marked in this figure. A total of 264 genes were derived from expert-reviewed panels for premature ovarian insufficiency (POI) and differences in sex development (DSD) available in Genomics England PanelApp (ENG; <https://panelapp.genomicsengland.co.uk/>) and PanelApp Australia (AUS; <https://panelapp-aus.org/>), encompassing varying levels of evidence (date accessed 10.01.2026). Of these, 157 genes were classified as high evidence (green), 55 as moderate evidence (amber), and 52 as low evidence (red), according to the PanelApp curation framework in which gene–disease relationships (GDR) are categorized based on the strength of available evidence (green = high/diagnostic-grade, amber = moderate, red = low/limited evidence). Additional genes were derived from the Online Mendelian Inheritance in Man (OMIM) database (https://www.omim.org/; date accessed 10.01.2026) and a systematic review published by Van Der Kelen *et al.*, 2023. Several genes overlapped across these sources.


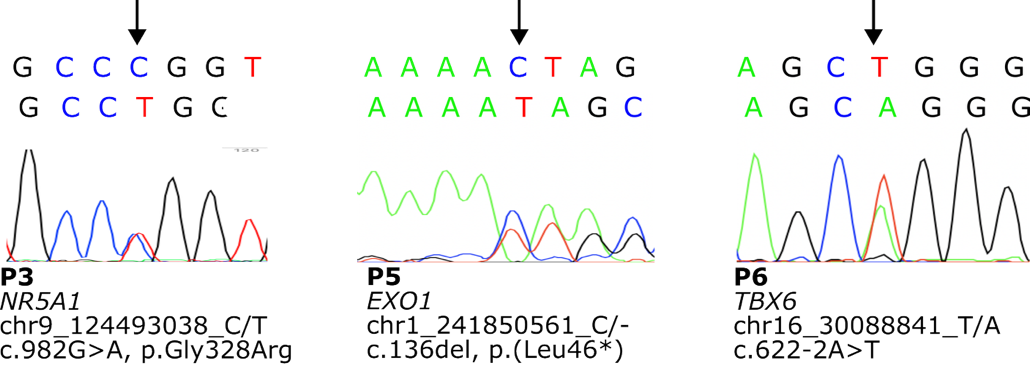


**Supplementary Figure S3. Chromatograms from Sanger sequencing validation of variants in P3, P5, P6.** Chromatograms for other patients with monogenic variants are presented in **Figure 1.** Primers for PCR and Sanger sequencing are provided in **Supplementary Table S6.**


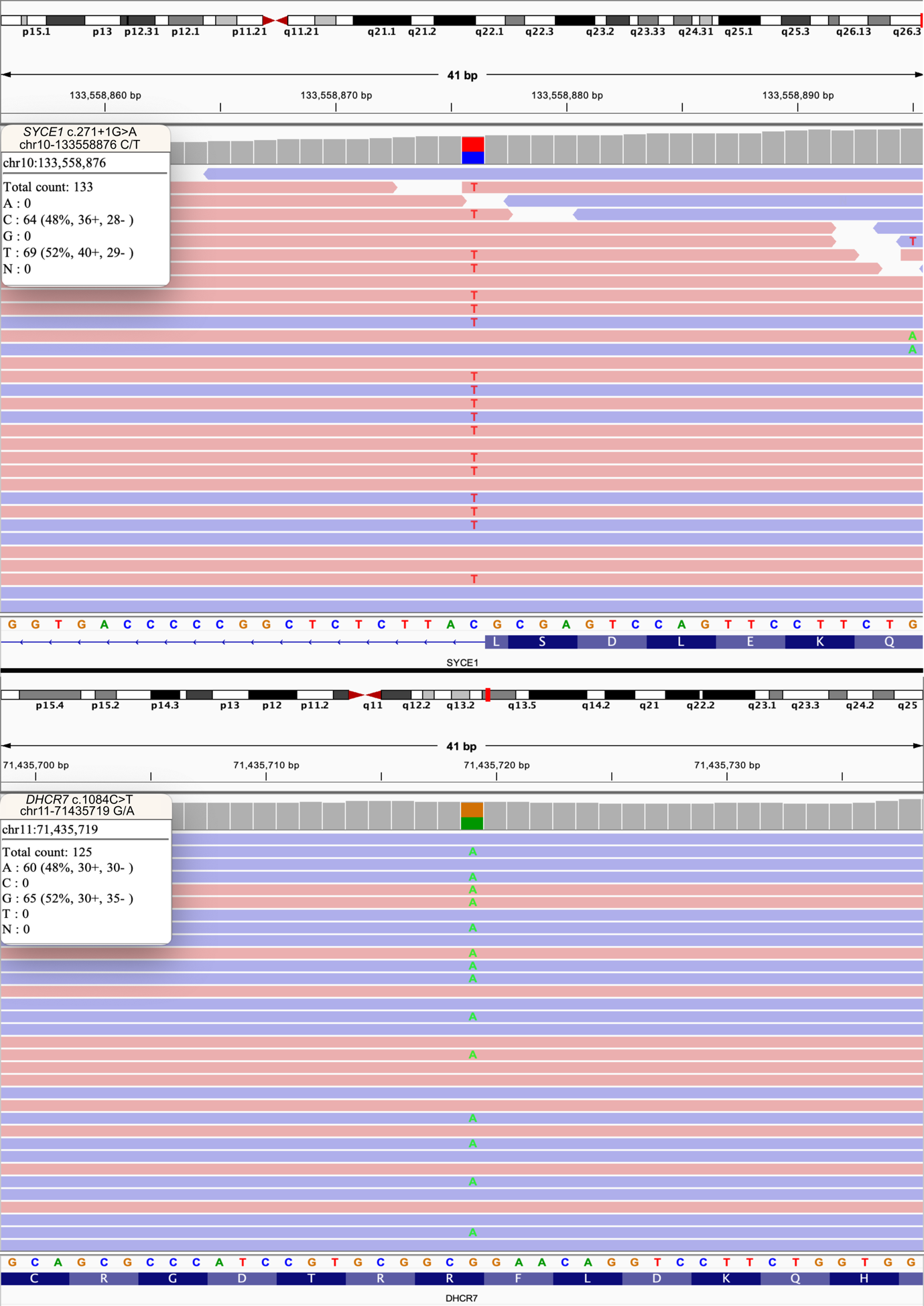
 **Supplementary Figure S4.** IGV visualization of heterozygous variants *SYCE1* c.271+1G>A (chr10-133558876 C/T) and *DHCR7* c.1084C>T (chr11-71435719 G/A). Both genes are linked to autosomal recessive (AR) conditions – premature ovarian insufficiency and Smith-Lemli-Opitz syndrome, respectively.

**
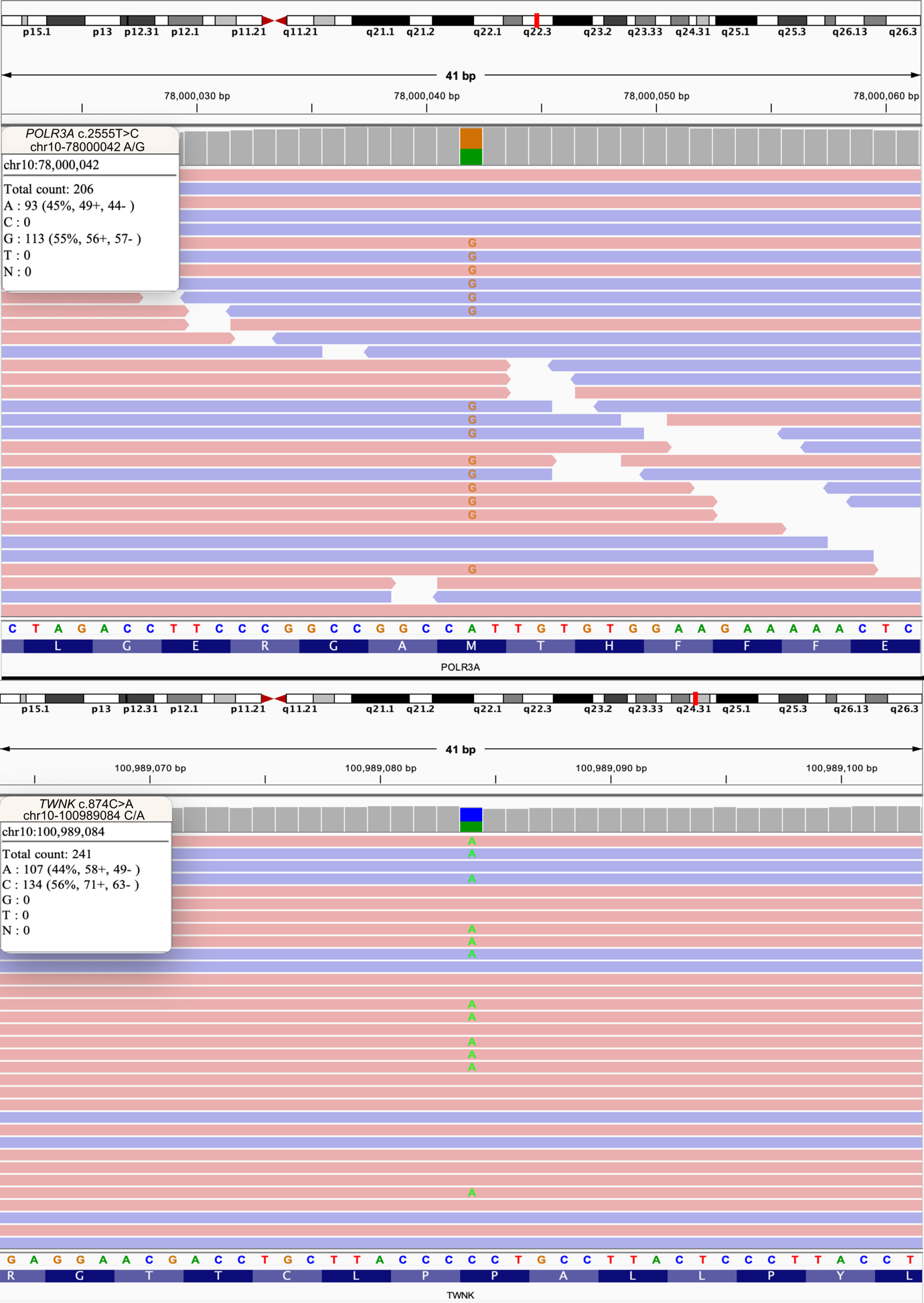
Supplementary Figure S5.** IGV visualization of heterozygous variants *POLR3A* c.2555T>C (chr10-78000042 A/G) and *TWNK* c.874C>A (chr10-100989084 C/A). Both genes are linked to autosomal recessive (AR) conditions – syndromic hypogonadotropic hypogonadism and Perrault syndrome, respectively.

**
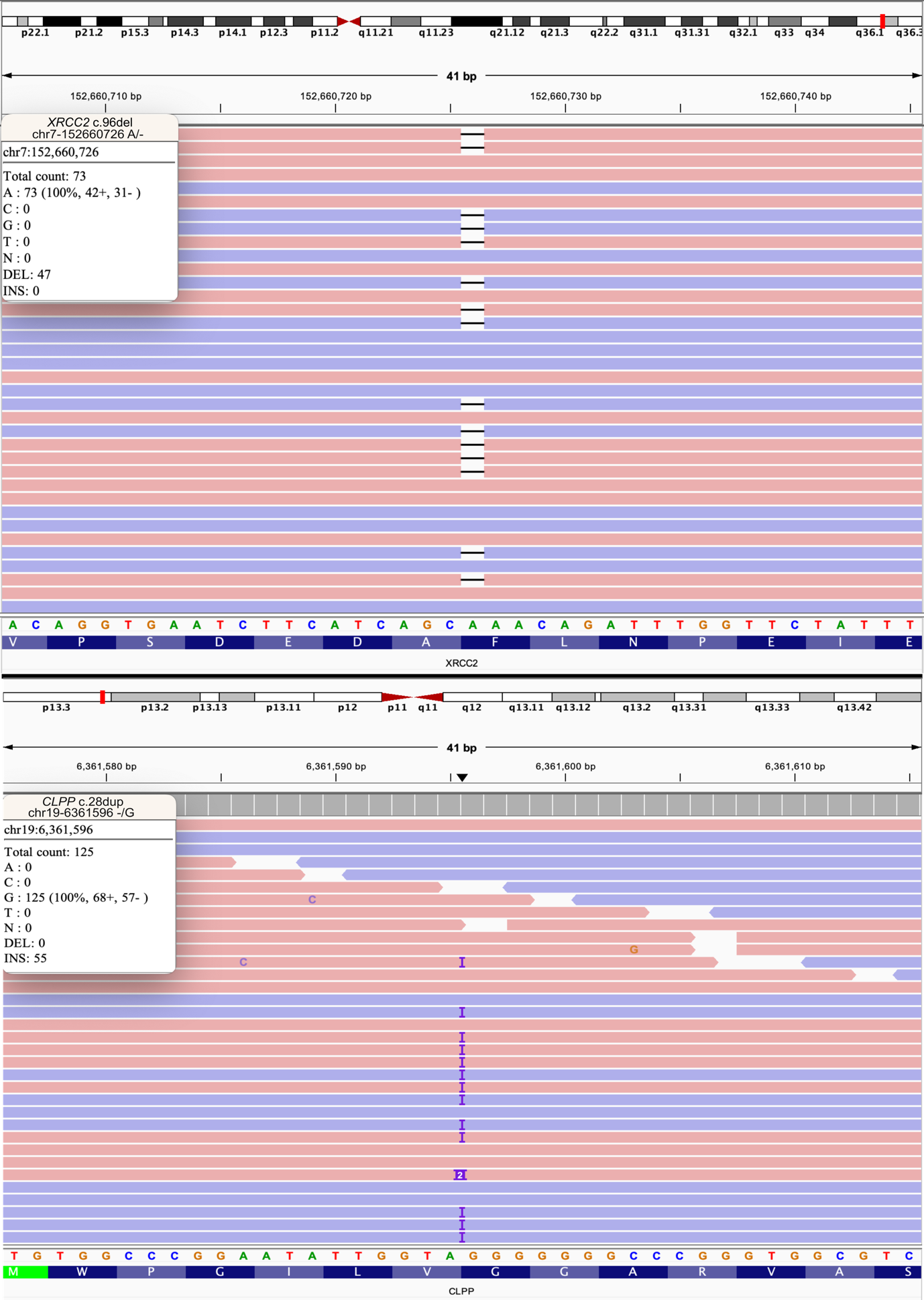
Supplementary Figure S6.** IGV visualization of heterozygous variants *XRCC2* c.96del (chr7-152660726 A/-) and *CLPP* c.28dup (chr19-6361596 -/G). Both genes are linked to autosomal recessive (AR) conditions – premature ovarian insufficiency, Fanconi anemia and spermatogenic failure (*XRCC2*) and Perrault syndrome (*CLPP*).

**
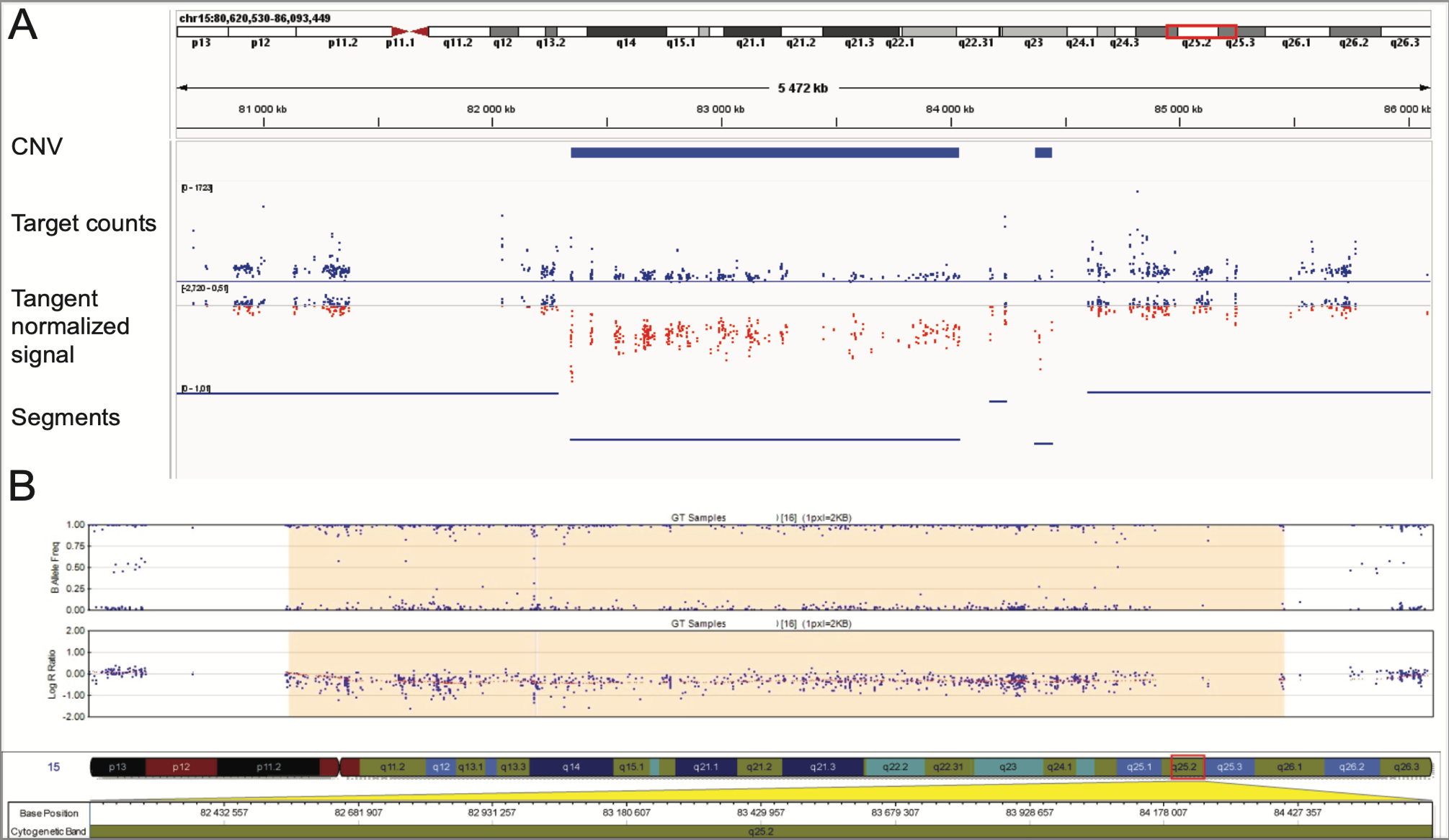
Supplementary Figure S7.** Visualization of 15q25.2 deletion (Chr15:82552258 - 84403399; 1.85 Mb) prediction from exome sequencing data (A image from Integrated Genome Viewer (Robinson et al., 2023)) and chromosomal microarray validation (B image from Genomestudio, Illumina Infinium Global Diversity Array-8 v1.0 BeadChip) for case P7. Detailed methods described in the Supplementary Materials and methods.

**
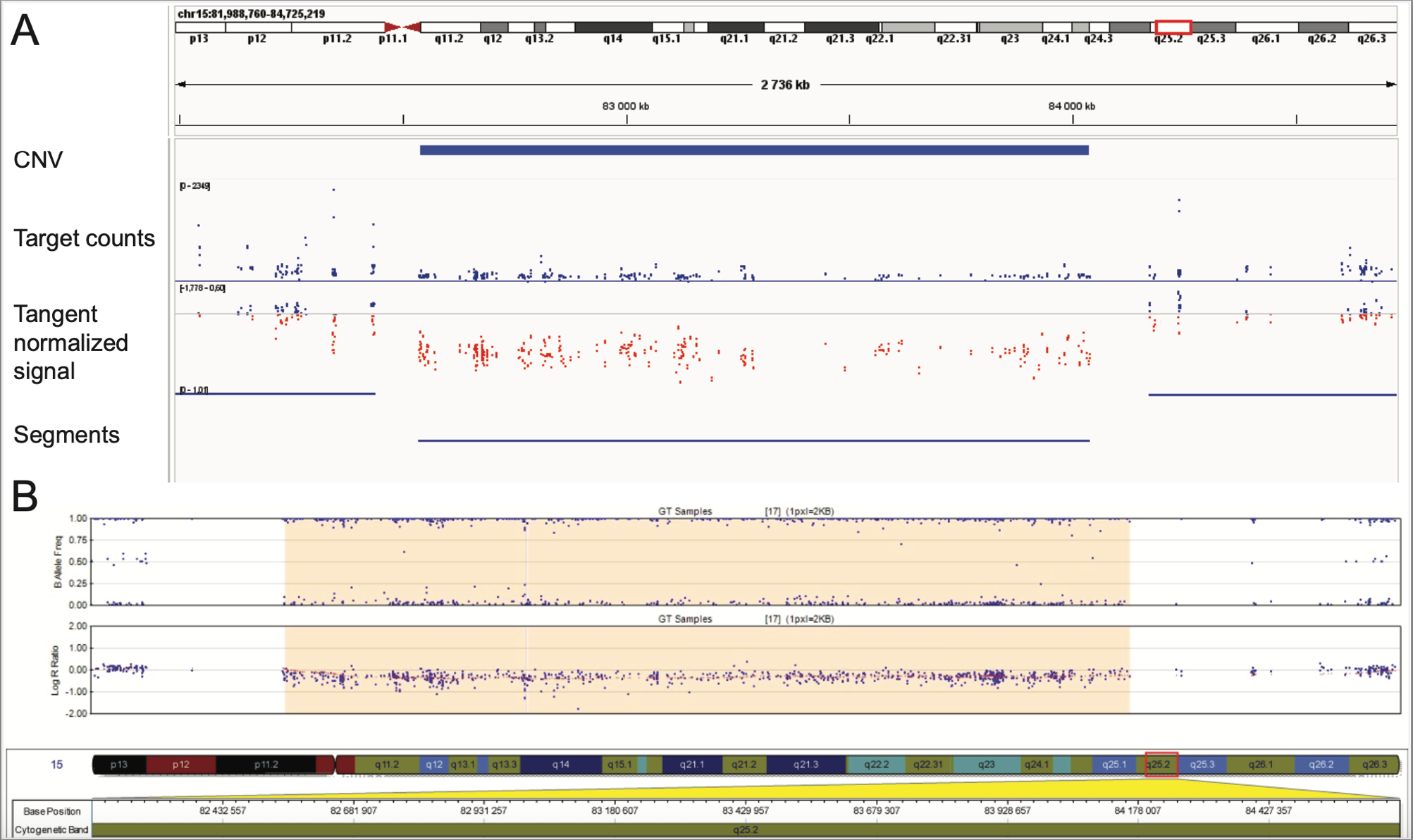
Suppementary Figure S8.** Visualization of 15q25.2 deletion (Chr15:82550590-84163390; 1.61 Mb) prediction from exome sequencing data (A image from Integrated Genome Viewer (Robinson *et al.*, 2023)) and chromosomal microarray validation (B image from Genomestudio, Illumina Infinium Global Diversity Array-8 v1.0 BeadChip) for case P8. Detailed methods described in Supplementary Materials and methods.

**Supplementary Figure S9.** Visualization of Xq27.3-Xq28 deletion (ChrX:147527872 – 156040896; 8.51 Mb) prediction from exome sequencing data (A, image from Integrated Genome Viewer (Robinson *et al.*, 2023)) and chromosomal microarray validation (B, image from Genomestudio, Illumina Infinium Global Diversity Array-8 v1.0 BeadChip) for case P9. Detailed methods are described in Supplementary Materials and methods.

**Supplementary Figure S10.** Visualization of 1q21.1 deletion (Chr1:146872717-148353326; 1.48 Mb) prediction from exome sequencing data (A image from Integrated Genome Viewer (Robinson *et al.*, 2023)) and chromosomal microarray validation (B image from Genomestudio, Illumina Infinium Global Diversity Array-8 v1.0 BeadChip) for case P10. Detailed methods described in Supplementary Materials and methods.

**
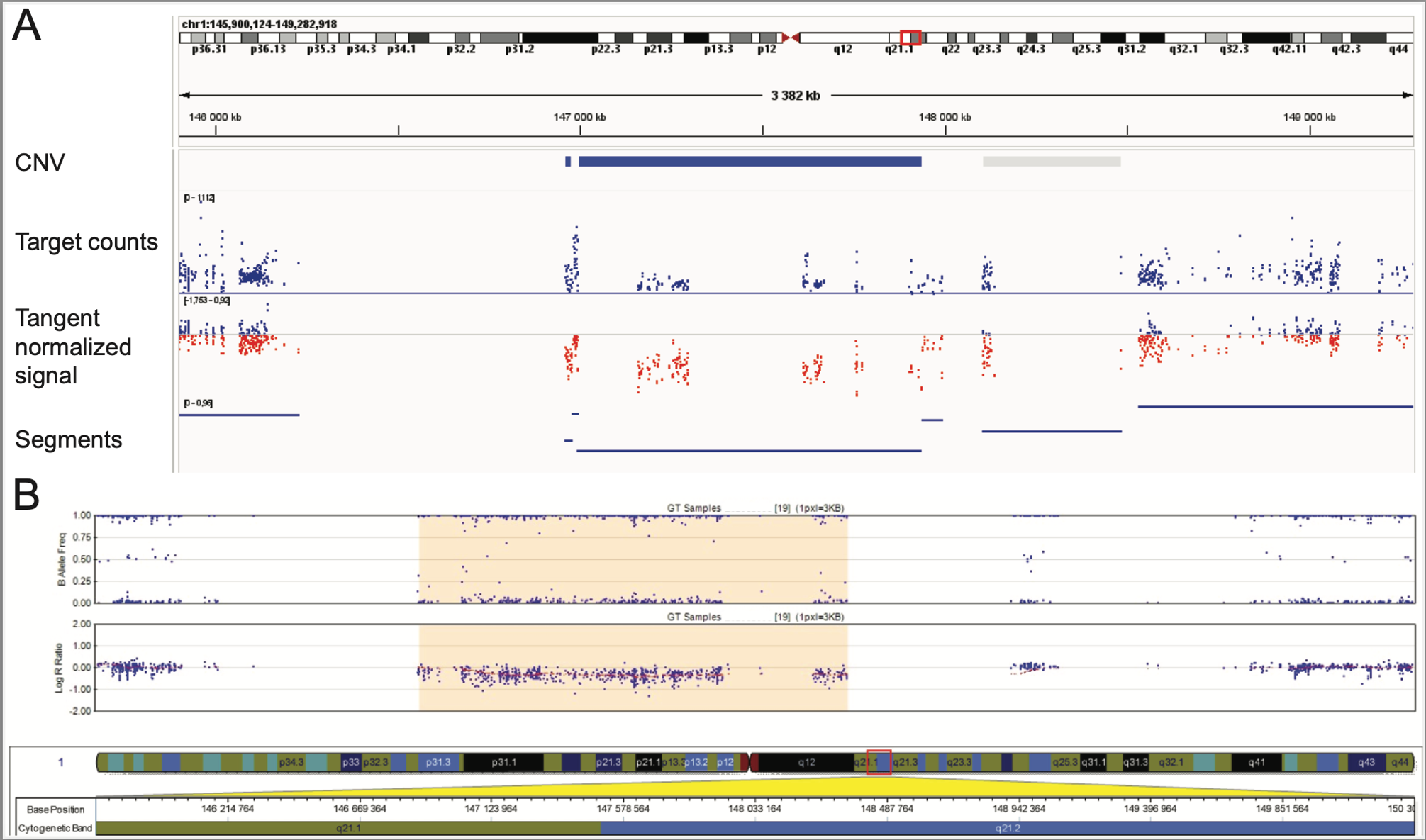
 Supplementary Figure S11.** Visualization of 1q21.1 deletion (Chr1:146872717-148353641; 1.48 Mb) prediction from exome sequencing data (A image from Integrated Genome Viewer (Robinson *et al.*, 2023)) and chromosomal microarray validation (B image from Genomestudio, Illumina Infinium Global Diversity Array-8 v1.0 BeadChip) for case P11. Detailed methods described in Supplementary Materials and methods.

**Supplementary Figure S12.** Visualisation of 1p36.22 duplication (Chr1:12273027-12776092; 0.5 Mb) prediction from exome sequencing data (A, image from Integrated Genome Viewer (Robinson *et al.*, 2023)) and chromosomal microarray validation (B, image from Genomestudio, Illumina Infinium Global Diversity Array-8 v1.0 BeadChip) for case P12. Detailed methods described in Supplementary Materials and methods.

**Supplementary Figure S13.** Visualisation of 12q21.1 duplication (Chr12:76430772-77942978; 1.51 Mb) prediction from exome sequencing data (A, image from Integrated Genome Viewer (Robinson *et al.*, 2023)) and chromosomal microarray validation (B, image from Genomestudio, Illumina Infinium Global Diversity Array-8 v1.0 BeadChip) for case P13. Detailed methods described in Supplementary Materials and methods.

# **SUPPLEMENTARY MATERIALS AND METHODS**

**Recruitment of participants and retrospective collection of health and family history.** All subjects were recruited and diagnosed by clinicians at the Women's Clinic of Tartu University Hospital (TUH) and the Women's Clinic and Centre of Endocrinology of East-Tallinn Central Hospital (ETCH).

Recruitment of patients followed two main approaches. First, doctors managing POI patients were informed of the study criteria, and patients were recruited through referrals. Patients who met the European Society of Human Reproduction and Embryology guideline (Panay *et al.*, 2024) criteria for idiopathic POI were referred to the study. FSH levels were obtained during routine clinical diagnosis/management. The exact timing relative to the menstrual cycle or diagnosis was not documented.

Secondly, laboratory data from 2012-2022 was sourced from both TUH and ETCH to identify patients with elevated FSH levels (>25 IU/L). For selected patients, clinical information was reviewed by their managing physician to determine whether they met the criteria for idiopathic POI. Upon patient consent, they were referred to the study.

All patients referred to the study underwent an interview to collect retrospective health and family data. Written informed consent for the evaluation and use of patients clinical data for scientific purposes, was obtained before the start of the interview. During the interview, personal health histories and family pedigrees were recorded, all following the same pipeline to ensure consistency.

**Biological Sample Collection, DNA Extraction, and Exome Sequencing (ES).** Following the interview, whole blood samples were collected at the respective clinic laboratories and stored at –80 °C until further processing. Genomic DNA was extracted using the QIAamp DNA Blood Maxi Kit (QIAGEN GmbH, Hilden, Germany), according to the manufacturer’s protocol. Exome Sequencing **(**ES) was performed using genomic DNA extracted from whole blood samples. Next Generation Sequencing (NGS) library preparation, sequencing, primary sequence analysis, and variant calling were performed in two sequencing centres: 40 DNA samples were sequenced at the NGS Service laboratory of the Institute for Molecular Medicine Finland (FIMM), Helsinki, Finland; 11 DNA samples at the accredited laboratory of Tartu University Hospital, Genetics and Personalized Medicine Clinic, Department of Laboratory Genetics, Tartu, Estonia.

**ES pipeline at the NGS Service Laboratory at FIMM, Helsinki, Finland.** 50 ng of gDNA was processed according to the Twist Human Core Exome EF Multiplex Complete kit (Twist Bioscience, San Francisco, CA, USA) manual. 4 µL of 15 µM Adapters used for ligation were unique dual index (UDI) oligos with unique molecular barcodes (UMI) by IDT (Integrated DNA Technologies, Coralville, IA, USA). Library quantification and quality control were performed using the LabChip GX Touch HT High Sensitivity assay (PerkinElmer, USA) and Qubit Broad Range DNA Assay (Thermo Fisher Scientific, Waltham, MA, USA). Libraries were pooled to 8-plex reactions according to concentration. The exome enrichment was performed using Twist Comprehensive Exome probes. The captured library pools were quantified for sequencing using KAPA Library Quantification Kit (KAPA Biosystems, Wilmington, MA, USA) and LabChip GX Touch HT High Sensitivity assay. Sequencing was performed with the Illumina NovaSeq system using S2 flow cell (Illumina, San Diego, CA, USA) and v1.5 chemistry. The read length for the paired-end run was 2×101 bp. Sequencing resulted in an average of 59 million reads per exome with an average median target coverage of 66X. Primary sequencing analysis and variant calling were performed using the Illumina DRAGEN Bio-IT Platform (v3.9 and v3.10, human genome build hg38).

**ES pipeline at the NGS Service Laboratory, TUH, Tartu, Estonia.** 600ng of gDNA was processed according to Illumina DNA Prep with Exome 2.5 Enrichment with Twist Bioscience for Illumina Mitochondrial Panel spike in kit according to the manufacturer's instructions. Library quantification and quality check were performed using Tapestation (Agilent, USA) and Qubit assays (Thermo Fisher Scientific, Waltham, MA, USA). Sequencing was performed with the Illumina NovaSeq X Plus system using a 10B flow cell (Illumina, San Diego, CA, USA). The read length for the paired-end run was 2×151 bp. Sequencing resulted in an average of 220 million reads per exome with an average coverage of 250x-350x, with 20x coverage over 99%. Primary sequencing analysis and variant calling were performed using the Illumina DRAGEN Bio-IT Platform (Dragen v4.1.23, Dragen Enrichment v.1.2.1, human genome build hg38). Sequencing resulted in an average of ~200 million reads per exome, with an average median target coverage of 267× (mean = 277×), 90% uniformity, and 7.9% of target bases covered ≥500×.

**Processing of VCF files and variant annotation using Variant Effect Predictor (VEP).** ES library preparation, data generation by NGS service laboratories and in-house bioinformatic pipeline for processing of primary sequencing data have been described in detail (Juchnewitsch *et al.*, 2024; Lillepea *et al.*, 2024; Valkna *et al.*, 2025). ES data generated from both sequencing centres were delivered as individual sample variant call format (VCF) files. All VCF files were filtered for quality using identical parameters. Variants with low depth of coverage (DP<10) and low genotype quality (GQ<20) were excluded. VCF files were further processed by filtering out heterozygous variants in the non-PAR regions of chromosome X (GRCh38 assembly, chrX:1-10000; chrX:2781480-155701382; chrX:156030896-156040895). Filtered VCF files of each sample were merged into a single VCF file and then segmented into individual chromosome files (chr1-22 and X) for variant annotation using Ensembl Variant Effect Predictor (VEP; v111) (McLaren *et al.*, 2016) in the offline mode and implementing the set of flags and plugins listed in **Supplementary Table S2.** Merging, filtering, and splitting of VCF files was performed with bcftools (v1.14) (Danecek *et al.*, 2021)

**Ploidy Estimation.** The sex karyotype of the samples was based on the ploidy estimator of the Illumina DRAGEN Bio-IT Platform (v3.9 and v3.10, GRCh38 assembly). The estimator first calculates the sequencing depth of coverage for each autosome and allosome in the human genome based on reads from the mapper/aligner. Then it estimates the sex karyotype of the sample by determining in which range, the ratios of the median sex chromosome coverages to the median autosomal coverage, falls in. All patients were estimated to be 46, XX.

**Kinship analysis.** The relatedness between the samples of patients with likely pathogenic /pathogenic (LP/P) variants was analyzed by the R (version 4.3.1) package SNPRelate (version 1.34.1). The Maximum Likelihood Estimation (MLE) for the Identity-By-Descent (IBD) analysis of the SNPRelate package was used for the analysis using maf = 0.05 and missing.rate = 0.05.

**Comparative population based cohort of pregnant women as a reference for population parameters in Figure 1B.** The comparative cohort of pregnant women (n=2334) illustrated in Figure 1B were recruited during their first antenatal visit at the Women's Clinic, Tartu University Hospital, in 2013 to 2015 without any preselection criteria. Detailed description of the recruitment process and and phenotyping strategies has been published previously (Kikas *et al.*, 2020). All participants were of white European ancestry and lived in Estonia. The project was approved by the Ethics Committee of Human Research of the University Clinic of Tartu, Estonia (permission no. 221/T-6, 17.12.2012, and 286/M-18, 15.10.2018) and was carried out in compliance with the Helsinki Declaration.

# **SUPPLEMENTARY FILE S1**

**Assessment of heterozygous P/LP variants in autosomal recessive genes.** No biallelic variants in autosomal or X-linked recessive genes were identified. However, six heterozygous P/LP variants in AR genes (*CLPP, DHCR7, POLR3A, TWNK, SYCE1, XRCC2*) were identified in six patients with secondary amenorrhea/oligomenorrhea (**Supplementary Table S11, Supplementary Figures S4-S6**). Unfiltered ES data for these cases were further examined to potentially identify a second P/LP variant that may have been excluded by the stringent variant-filtering pipeline. No second P/LP variant was found in these genes. According to the Genomic England PanelApp (<https://panelapp.genomicsengland.co.uk>), the PanelApp Australia (<https://panelapp-aus.org>), and Online Mendelian Inheritance in Man (OMIM, <https://www.omim.org/>), all these genes, except *TWNK,* are implicated solely in biallelic (recessive) conditions. It was concluded that there is a lack of evidence that heterozygous findings in *CLPP, DHCR7, POLR3A, SYCE1* or *XRCC2* could be causatively linked to POI in our patients.

Although all *TWNK*-related reproductive conditions have been classified as recessive, some *TWNK*-linked diseases have been shown to exhibit monoallelic inheritance. Therefore, TWNK p.(Pro292Thr) identified in a secondary amenorrhea case in our cohort was investigated further. In the literature, this variant has been reported as disease-causing only in homozygous individuals, including in a case of Perrault syndrome (Hu *et al.*, 2019; Jamali *et al.*, 2019; Shokouhian *et al.*, 2026). In the reported families, heterozygous female carriers of P/LP variants presented with an unaffected phenotype (Shokouhian *et al.*, 2026). Additionally, a recent study has shown that 46,XX individuals with heterozygous *TWNK* loss-of-function variants does not present POI and TWNK haploinsufficiency exhibits only a modest shift toward earlier age at menopause (mean 49 yrs; ~1.54 yrs earlier than in the reference cohort) (Shekari *et al.*, 2023). Supported by these data, we concluded that heterozygous P/LP variants in *TWNK* are not causatively linked to POI.

**Identification of an independent male patient of NR2F2 p.(Val307Ala) variant (Case A1).** Additional infertile subject with NR2F2 p.(Val307Ala) variant was identified from the ES dataset of the ESTonian ANDrology (ESTAND) cohort, collected with the aim to identify genetic causes of male inferility (Ehala-Aleksejev and Punab, 2015; Punab *et al.*, 2017) (clinical research PI: M. Punab, genetic research PI: M. Laan). All ESTAND participants have been recruited at the Andrology Clinic of Tartu University Hospital, Tartu, Estonia. Detailed recruitment and andrological phenotyping protocol has been described recently (Juchnewitsch *et al.*, 2024; Lillepea *et al.*, 2024). Genetic research of the ESTAND participants was approved by the Ethics Review Committee of Human Research of the University of Tartu, Estonia (permission no. 404/T-24).

*NR2F2* c.920T>C [p.(Val307Ala)] was identified in a 33-years-old Estonian oligozoospermia case in an independent research project focusing on monogenic male infertility (*O.Mõttus, M.Laan,* *unpublished*). ES data for this subject had been generated at the NGS Service Laboratory at TUH, Tartu, Estonia (*see above*).

The patient presented low sperm counts (total sperm count 14,7 million/ ejaculate; reference > 39 million/ejaculate, (“WHO laboratory manual for the examination and processing of human semen,” n.d.) and unilateral cryptorchidism, resolved by orchidopexy at a young age. His hormone levels were within reference range – FSH 10.9 IU/l; LH 8,04 IU/L and testosterone 11.4 nmol/L (reference values are based on Tartu University Hospital, United Laboratories reference values available in https://www.kliinikum.ee/yhendlabor/kasiraamat/). His son had normal testicular descent and was confirmed to carry the wildtype TT-genotype (**Figure 1D**).

**REFERENCES TO SUPPLEMENTARY MATERIALS AND METHODS**

Danecek P, Bonfield JK, Liddle J, Marshall J, Ohan V, Pollard MO, Whitwham A, Keane T, McCarthy SA, Davies RM, *et al.* Twelve years of SAMtools and BCFtools. *GigaScience* 2021;**10**:giab008.

Ehala-Aleksejev K, Punab M. The different surrogate measures of adiposity in relation to semen quality and serum reproductive hormone levels among Estonian fertile men. *Andrology* 2015;**3**:225–234.

Hu H, Kahrizi K, Musante L, Fattahi Z, Herwig R, Hosseini M, Oppitz C, Abedini SS, Suckow V, Larti F, *et al.* Genetics of intellectual disability in consanguineous families. *Mol Psychiatry* 2019;**24**:1027–1039.

Jamali F, Ghaedi H, Tafakhori A, Alehabib E, Chapi M, Daftarian N, Darvish H, Jamshidi J. Homozygous Mutation in TWNK Cases Ataxia, Sensorineural Hearing Loss and Optic Nerve Atrophy. *Arch Iran Med* 2019;**22**:728–730.

Juchnewitsch A-G, Pomm K, Dutta A, Tamp E, Valkna A, Lillepea K, Mahyari E, Tjagur S, Belova G, Kübarsepp V, *et al.* Undiagnosed RASopathies in infertile men. *Front Endocrinol* 2024;**15**:1312357.

Kikas T, Dutta A, Inno R, Pomm K, Tjagur S, Poolamets O, Roomere H, Punab M, Laan M. Microdeletion and microduplication syndromes, including recurrent rearrangements at 16p11.2 and 22q11.21, are enriched in unexplained male infertility. *Hum Reprod* 2025;deaf231.

Kikas T, Inno R, Ratnik K, Rull K, Laan M. C-allele of rs4769613 Near FLT1 Represents a High-Confidence Placental Risk Factor for Preeclampsia. *Hypertension* 2020;**76**:884–891.

Lillepea K, Juchnewitsch A-G, Kasak L, Valkna A, Dutta A, Pomm K, Poolamets O, Nagirnaja L, Tamp E, Mahyari E, *et al.* Toward clinical exomes in diagnostics and management of male infertility. *Am J Hum Genet* 2024;**111**:877–895.

McLaren W, Gil L, Hunt SE, Riat HS, Ritchie GRS, Thormann A, Flicek P, Cunningham F. The Ensembl Variant Effect Predictor. *Genome Biol* 2016;**17**:122.

Panay N, Anderson RA, Bennie A, Cedars M, Davies M, Ee C, Gravholt CH, Kalantaridou S, Kallen A, Kim KQ, *et al.* Evidence-based guideline: premature ovarian insufficiency†,‡. *Hum Reprod Open* 2024;**2024**:hoae065.

Punab M, Poolamets O, Paju P, Vihljajev V, Pomm K, Ladva R, Korrovits P, Laan M. Causes of male infertility: a 9-year prospective monocentre study on 1737 patients with reduced total sperm counts. *Hum Reprod* 2017;**32**:18–31.

Richards S, Aziz N, Bale S, Bick D, Das S, Gastier-Foster J, Grody WW, Hegde M, Lyon E, Spector E, *et al.* Standards and guidelines for the interpretation of sequence variants: a joint consensus recommendation of the American College of Medical Genetics and Genomics and the Association for Molecular Pathology. *Genet Med Off J Am Coll Med Genet* 2015;**17**:405–424.

Robinson JT, Thorvaldsdottir H, Turner D, Mesirov JP. igv.js: an embeddable JavaScript implementation of the Integrative Genomics Viewer (IGV). *Bioinforma Oxf Engl* 2023;**39**:btac830.

Shekari S, Stankovic S, Gardner EJ, Hawkes G, Kentistou KA, Beaumont RN, Mörseburg A, Wood AR, Prague JK, Mishra GD, *et al.* Penetrance of pathogenic genetic variants associated with premature ovarian insufficiency. *Nat Med* 2023;**29**:1692–1699.

Shokouhian E, Kahrizi K, Najmabadi H, Babanejad M. Genetic etiology of Perrault syndrome in Iranian families: first report from Iran and literature review. *J Appl Genet* 2026;**67**:127–137.

Valkna A, Juchnewitsch A-G, Põlluaas L, Lillepea K, Tjagur S, Dutta A, Pomm K, Punab M, Laan M. Significantly increased load of hereditary cancer-linked germline variants in infertile men. *Hum Reprod Open* 2025;**2025**:hoaf008.

Van Der Kelen A, Okutman Ö, Javey E, Serdarogullari M, Janssens C, Ghosh MS, Dequeker BJH, Perold F, Kastner C, Kieffer E, *et al.* A systematic review and evidence assessment of monogenic gene–disease relationships in human female infertility and differences in sex development. *Hum Reprod Update* 2023;**29**:218–232. Oxford Academic.

WHO laboratory manual for the examination and processing of human semen. Available from: https://www.who.int/publications-detail-redirect/9789240030787.
