# Supplementary material for: All-in-one exome sequencing approach for genetic testing of unexplained premature ovarian insufficiency
Source: Hum Reprod Open. 2026 Jun 17;2026(3):hoag058. doi: 10.1093/hropen/hoag058 (PMC13372669; doi:10.1093/hropen/hoag058)
Supplement: hoag058_Supplementary_Data [file hoag058_supplementary_data.zip › Supplementary File S1.pdf]

SUPPLEMENTARY FILE S1

Assessment of heterozygous P/LP variants in autosomal recessive genes. No biallelic variants in autosomal or X-linked recessive genes were identified. However, six heterozygous P/LP variants in AR genes (*CLPP, DHCR7, POLR3A, TWNK, SYCE1, XRCC2*) were identified in six patients with secondary amenorrhea/oligomenorrhea (**Supplementary Table S11, Supplementary Figures S4-S6**). Unfiltered ES data for these cases were further examined to potentially identify a second P/LP variant that may have been excluded by the stringent variant-filtering pipeline. No second P/LP variant was found in these genes. According to the Genomic England PanelApp (https://panelapp.genomicsengland.co.uk), the PanelApp Australia (https://panelapp-aus.org), and Online Mendelian Inheritance in Man (OMIM, https://www.omim.org/), all these genes, except *TWNK,* are implicated solely in biallelic (recessive) conditions. It was concluded that there is a lack of evidence that heterozygous findings in *CLPP, DHCR7, POLR3A, SYCE1* or *XRCC2* could be causatively linked to POI in our patients.

Although all *TWNK*-related reproductive conditions have been classified as recessive, some *TWNK*-linked diseases have been shown to exhibit monoallelic inheritance. Therefore, TWNK p.(Pro292Thr) identified in a secondary amenorrhea case in our cohort was investigated further. In the literature, this variant has been reported as disease-causing only in homozygous individuals, including in a case of Perrault syndrome (Hu *et al.*, 2019; Jamali *et al.*, 2019; Shokouhian *et al.*, 2026). In the reported families, heterozygous female carriers of P/LP variants presented with an unaffected phenotype (Shokouhian *et al.*, 2026). Additionally, a recent study has shown that 46,XX individuals with heterozygous *TWNK* loss-of-function variants does not present POI and TWNK haploinsufficiency exhibits only a modest shift toward earlier age at menopause (mean 49 yrs; ~1.54 yrs earlier than in the reference cohort) (Shekari *et al.*, 2023). Supported by these data, we concluded that heterozygous P/LP variants in *TWNK* are not causatively linked to POI.

Identification of an independent male patient of NR2F2 p.(Val307Ala) variant (Case A1). Additional infertile subject with NR2F2 p.(Val307Ala) variant was identified from the ES dataset of the ESTonian ANDrology (ESTAND) cohort, collected with the aim to identify genetic causes of male inferility (Ehala-Aleksejev and Punab, 2015; Punab *et al.*, 2017) (clinical research PI: M. Punab, genetic research PI: M. Laan). All ESTAND participants have been recruited at the Andrology Clinic of Tartu University Hospital, Tartu, Estonia. Detailed recruitment and andrological phenotyping protocol has been described recently (Juchnewitsch *et al.*, 2024; Lillepea *et al.*, 2024). Genetic research of the ESTAND participants was approved by the Ethics Review Committee of Human Research of the University of Tartu, Estonia (permission no. 404/T-24).

NR2F2 c.920T>C [p.(Val307Ala)] was identified in a 33-years-old Estonian oligozoospermia case in an independent research project focusing on monogenic male infertility (*O.Mõttus, M.Laan, unpublished*). ES data for this subject had been generated at the NGS Service Laboratory at TUH, Tartu, Estonia (*see above*).

The patient presented low sperm counts (total sperm count 14,7 million/ ejaculate; reference > 39 million/ejaculate, (World Health Organization, 2021) and unilateral cryptorchidism, resolved by orchidopexy at a young age. His hormone levels were within reference range – FSH 10.9 IU/l; LH 8,04 IU/L and testosterone 11.4 nmol/L (reference values are based on Tartu University Hospital, United Laboratories reference values available in https://www.kliinikum.ee/yhendlabor/kasiraamat/). His son had normal testicular descent and was confirmed to carry the wildtype TT-genotype (**Figure 1D**).

REFERENCES TO SUPPLEMENTARY FILE S1

Ehala-Aleksejev K, Punab M. The different surrogate measures of adiposity in relation to semen quality and serum reproductive hormone levels among Estonian fertile men. *Andrology* 2015;**3**:225–234.

Hu H, Kahrizi K, Musante L, Fattahi Z, Herwig R, Hosseini M, Oppitz C, Abedini SS, Suckow V, Larti F, *et al.* Genetics of intellectual disability in consanguineous families. *Mol Psychiatry* 2019;**24**:1027–1039.

Jamali F, Ghaedi H, Tafakhori A, Alehabib E, Chapi M, Daftarian N, Darvish H, Jamshidi J. Homozygous Mutation in TWNK Cases Ataxia, Sensorineural Hearing Loss and Optic Nerve Atrophy. *Arch Iran Med* 2019;**22**:728–730. Academy of Medical Sciences of I.R. Iran.

Juchnewitsch A-G, Pomm K, Dutta A, Tamp E, Valkna A, Lillepea K, Mahyari E, Tjagur S, Belova G, Kübarsepp V, *et al.* Undiagnosed RASopathies in infertile men. *Front Endocrinol* 2024;**15**:1312357.

Lillepea K, Juchnewitsch A-G, Kasak L, Valkna A, Dutta A, Pomm K, Poolamets O, Nagirnaja L, Tamp E, Mahyari E, *et al.* Toward clinical exomes in diagnostics and management of male infertility. *Am J Hum Genet* 2024;**111**:877–895.

Punab M, Poolamets O, Paju P, Vihljajev V, Pomm K, Ladva R, Korrovits P, Laan M. Causes of male infertility: a 9-year prospective monocentre study on 1737 patients with reduced total sperm counts. *Hum Reprod* 2017;**32**:18–31.

Shekari S, Stankovic S, Gardner EJ, Hawkes G, Kentistou KA, Beaumont RN, Mörseburg A, Wood AR, Prague JK, Mishra GD, *et al.* Penetrance of pathogenic genetic variants associated with premature ovarian insufficiency. *Nat Med* 2023;**29**:1692–1699.

Shokouhian E, Kahrizi K, Najmabadi H, Babanejad M. Genetic etiology of Perrault syndrome in Iranian families: first report from Iran and literature review. *J Appl Genet* 2026;**67**:127–137.

World Health Organization. *WHO laboratory manual for the examination and processing of human semen Sixth Edition* 2021..
